# RELT Is Upregulated in Breast Cancer and Induces Death in Breast Cancer Cells

**DOI:** 10.3390/biomedicines12122667

**Published:** 2024-11-22

**Authors:** Maryann Batiste, Bethany Joy, Cara K. Yee, Luke Cho, Ashley Christensen, Ihab Abed, Kailey Nguyen, Anusri Yanumula, Hannah Chang, Evan D. Cho, Wenjia Wang, Emily Chou, Esther H. Chang, Yennie L. Shyu, Alyssa Abram, Jessa Alcaide, James Zhou, Brittany Gillespie, Michelle Senderovich, Gianne Almeida Cusick, Ai-Vy Le, Frank Hoang, Yihui Shi, Eslam Mohamed, John K. Cusick

**Affiliations:** 1 Department of Basic Science, College of Medicine, California Northstate University, Elk Grove, CA 95757, USAbethany.joy7985@cnsu.edu (B.J.); ashley.christensen9832@cnsu.edu (A.C.); hannah.chang10229@cnsu.edu (H.C.); evan.cho10275@cnsu.edu (E.D.C.); esther.chang8491@cnsu.edu (E.H.C.); yennie.shyu@ucsf.edu (Y.L.S.); aivy.minhle@gmail.com (A.-V.L.); yihui.shi@sutterhealth.org (Y.S.); eslam.mohamed@cnsu.edu (E.M.); 2 Masters of Pharmaceutical Sciences Department, College of Graduate Studies, California Northstate University, Elk Grove, CA 95757, USA; 3 California Pacific Medical Center Research Institute, San Francisco, CA 94107, USA

**Keywords:** RELT, TNFRSF19L, RELL1, RELL2, tumor necrosis factor receptor, OXSR1 kinase, breast cancer, apoptosis, caspase

## Abstract

Background: Receptor Expressed in Lymphoid Tissues (RELT) is a TNFRSF member that has two paralogs, RELL1 and RELL2; the three proteins are collectively referred to as RELT family members (RELTfms). Methods: We sought to evaluate RELT expression in cancerous cells by using real-time PCR, western blotting, flow cytometry, and immunohistochemistry (IHC). The mechanism of RELT-induced cell death was assessed by western blotting, flow cytometry, luciferase assays, and morphology staining. RELT localization was detected through immunofluorescence and western blotting, and co-immunoprecipitation was used to test whether a mutated RELT interacts with the OXSR1 kinase. Results: RELT and RELL1 protein expression was significantly elevated in cell lines representing breast and lung cancer, whereas RELL2 protein expression was relatively consistent across different cell lines. The surface expression of RELT was highest in monocytes. IHC staining revealed increased RELT expression in malignant breast cancer biopsies compared to patient-matched benign tissue. RELTfm overexpression induced death in MDA-MB-231 (231) breast cancer cells, accompanied by increased phosphatidylserine externalization and Caspase-3/7 activation. The co-transfection of plasmids predicted to block the phosphorylation of RELT by the OXSR1 kinase did not abrogate RELT-induced apoptosis, indicating that the activation of p38 by RELT through the OXSR1 kinase is not required for RELT-induced cell death. Interestingly, nuclear localization of RELT was detected in 231 and HEK-293 cells. Conclusions: These results demonstrate that RELT induces death in breast cancer cells through an apoptotic pathway that does not require OXSR1 phosphorylation and that RELT possesses the ability to translocate to the nucleus, a novel finding that warrants further investigation.

## 1. Introduction

Tumor Necrosis Factor Receptor Superfamily (TNFRSF) members are signaling proteins that govern many processes, such as differentiation, proliferation, and apoptosis [1]. TNFRSF members predominantly function by initiating signal transduction pathways in response to binding soluble or membrane-bound ligands of the Tumor Necrosis Factor Superfamily (TNFSF). There are currently 29 identified TNFRSF members and 19 TNFSF members. Homologs of these proteins exist in invertebrates, yet it is believed that both families expanded by duplication and divergence during the creation of the adaptive immune system in vertebrates [2]. Although intimately associated with the governing of the immune system, TNFRSF and TNFSF members also play critical roles in non-immune cells and tissues, and mutations in these signaling pathways can result in many diseases associated with organogenesis, development, inflammation, autoimmunity, and cancer [3].

RELT (Receptor Expressed in Lymphoid Tissues), also known as TNFRSF19L, is a TNFRSF member implicated as an important regulator of the immune system and is associated with several cancers [4]. The ligand for RELT is unknown, and there is relatively little known about this protein in comparison to other TNFRSF members. RELT was named for its high levels of mRNA expression in the hematopoietic system [5], and mice lacking RELT exhibited an increased level of T-cell activation, implicating RELT as a protein that normally functions to inhibit the activation of T-cells [6]. Additionally, several groups have demonstrated that RELT is essential for the proper formation of enamel [7,8], indicating that RELT serves additional functions independent of the immune system. RELL1 and RELL2 (RELT Like 1 and 2) are paralogs of RELT that physically bind and co-localize with each other, as well as RELT, at the plasma membrane [9]; these three proteins are collectively referred to as RELT family members (RELTfms) [4]. RELTfms induce apoptosis in HEK-293 (293) epithelial cells [10], and the ability of RELT to induce apoptosis in 293 cells is independent of Fadd and Caspase-8 [11]. RELTfms activate the p38 pathway in a manner that is dependent on the phosphorylation of RELT family members by the closely related kinases OXSR1 (also known as OSR1 for Oxidative Stress Responsive Kinase 1) and SPAK (STE20/SPS1-related proline/alanine-rich kinase) [11,12]. RELL1 and RELL2 lack extensive extracellular sequences and are hypothesized to serve as adaptor proteins that recruit unique signal-transducing partners to RELT [9], yet RELL1 and RELL2 likely possess functions independent of RELT, especially for RELL1, considering it is abundantly expressed in tissues lacking RELT expression [4,9]. RELL1 inhibits autophagy in macrophages, and RELL1 expression is correlated with *M. tuberculosis* survival in macrophages [13]. The most dramatic phenotype reported of mice lacking RELL1 was in behavior and auditory processing, as reported by the International Mouse Phenotyping Consortium, www.mousephenotype.org [14] (accessed on 25 September 2024). A mouse knockout phenotype for RELL2 has not yet been reported. Interestingly, functional attributes have been discovered for RNA molecules transcribed from RELTfm genes. Distinct circular RNA molecules expressed from the RELL1 promoter exert a pro-inflammatory effect on endothelial cells [15], promote inflammatory osteoarthritis progression [16], and inhibit autophagy [17]. Likewise, a long non-coding RNA molecule expressed from the RELL2 gene was identified as a poor prognostic indicator for intrahepatic cholangiocarcinoma [18]. These results emphasize the need for considering the activities of both RNA molecules and proteins to gain a comprehensive understanding regarding the function of individual RELTfm genes.

Increasing evidence strongly ties RELTfms with several cancers [4]. RELT overexpression promotes cell cycle progression in esophageal squamous cell carcinoma (ESCC) through activation of NF-κB and inhibition of p27 and Caspase-3 [19]. RELT also promotes bone lesions in multiple myeloma cells through the activation of NF-κB [20]. RELT is upregulated in mouse lung tumors [21] and in human B-cell lymphomas [22]. Bioinformatic analysis indicates that RELT is a poor prognostic indicator for renal cancer [23], head and neck cancer [24], and prostate cancer [25,26]. Conversely, bioinformatic analysis predicts that RELT is protective against small-cell lung cancer [27]. Bioinformatic analysis indicates that RELL1 expression is a poor prognostic indicator for gliomas [28] and may serve a pro-tumorigenic role in colorectal cancer [29]. Conversely, a circular RNA molecule expressed from the RELL1 gene inhibits gastric cancer [17]. Bioinformatic analysis indicates that RELL2 is upregulated and is a poor prognostic indicator for several cancers [30], and a separate study identified RELL2 as a poor prognostic indicator for acute myeloid leukemia [31]. However, both ex vivo and in vivo evidence indicates that RELL2 expression induces apoptosis in ESCC, and alternative exon selection determines whether a functional anti-tumorigenic RELL2 protein is synthesized to exert an anti-tumorigenic effect [32]. Similarly, RELL2 exhibited an anti-tumorigenic effect on pancreatic ductal adenocarcinoma cells in a manner that was dependent on the presence of the RNA splicing factor DHX38 [33].

We sought to further explore the relationship between RELT and cancer by examining the expression of RELT family members in various cancer cell lines using Western blotting. Unexpectedly, RELT protein was expressed at higher levels in breast cancer (BC) cell lines in comparison to cell lines of the hematopoietic system. We therefore sought to examine the relationship between RELTfms and BC more closely. This study examined the ability of RELTfms to induce death in BC cells and examined the expression of RELT in biopsies of human BC. We report that RELTfms induce apoptosis in MDA-MB-231 (231) cells by an apoptotic pathway and report that RELT expression is altered and upregulated in biopsies of human BC. Finally, we examined the cellular localization of RELT and reported that RELT associates with membranes and the cytoskeleton and can also migrate to the nucleus.

## 2. Materials and Methods

DNA constructs. The expression plasmid constructs for HA-RELT, HA-RELL1, Flag-RELL1, HA-RELL2, Flag-RELL2, Flag-OXSR1 (OSR1), and the Flag-OXSR1 K46M mutant construct were described previously [9,22]. For RELT mutagenesis, the primers 5′-GTCTGTGGGCAGGGCCCGCGCGGCTCGAATTCCTG-3′ and 5′ CAGGAATTCGAGCCGCGCGGGCCCTGCCCACAGAC-3′ were purchased from Integrated DNA technologies and utilized to create an HA-RELT construct with an RFRV → RARA mutation at amino acids 349–352 using a site mutagenesis kit (Agilent, cat: 210513). DNA sequencing was performed to confirm the presence of the introduced mutation and the absence of any additional unexpected mutations in the modified RELT gene.

Cell Culture and transfections. Seven cell lines were purchased from ATCC and cultured with complete culture media corresponding to the appropriate cell type as follows: MCF7 (breast cancer cell line) was cultured in Eagle’s Minimum Essential Medium (ATCC, cat: 30-2003) with 0.01 mg/mL of insulin, 10% FBS, and 1% Penicillin–Streptomycin mix; H-358 (lung cancer cell line), Jurkat (T-cell leukemia cell line), Raji (B-cell lymphoma cell line), and Thp-1 (monocyte cell line) were cultured in RPMI 1640 (Corning, cat: 10-040-CV) with 10% FBS and 1% Penicillin–Streptomycin mix; HEK-293 (embryonic kidney epithelial cell line) and the triple-negative breast cancer cell line MDA-MB-231 (231) were cultured in DMEM (Corning, cat: 10-013-CV) with 10% FBS and 1% Penicillin–Streptomycin mix. All cells were maintained in a Binder C150 UL CO_2_ incubator at 37 °C and 5% CO_2_. For transient transfections of DNA plasmids, lipofectamine 3000 reagent (ThermoFisher Scientific, cat: L300008) was used to transfect HEK-293 cells according to the manufacturer’s instructions. Similarly, TransfeX transfection reagent (ATCC, cat: ACS-4005) was used to transfect 231 cells according to the manufacturer’s recommendations.

RNA extraction, cDNA synthesis, and Real-Time PCR. Single-cell suspensions were prepared for each cell line (see above). RNA was extracted from 1 million cells for each cell line per the manufacturer’s instructions using Ambion TRIZOL^®^ Reagent (ThermoFisher Scientific, cat: 15596026). RNA was quantified using a NanoDrop 2000c Spectrophotometer. A total of 1.5 µg of RNA was used for cDNA synthesis. The cDNA synthesis was performed per the manufacturer’s instructions using High-Capacity cDNA Reverse Transcription Kit (Applied Biosystems, cat: 4368814). Real Time PCR (RT-PCR) was performed per the manufacturer’s specifications using qPCRBIO SyGreen Blue Mix Lo-ROX (Genesee Scientific, cat: 17-505B). Samples were loaded in triplicates in a 96-well PCR plate, and the RT-PCR was performed using CFX ConnectTM Real-Time System. β-actin was used as the housekeeping gene for normalization. The relative expression of the targets was calculated using ^∆∆CT^ method. Primer sequences were as follows:

β-actin-Fwd: 5′-GACGACATGGAGAAAATCTG-3′.

β-actin-Rev: 5′-ATGATCTGGGTCATCTTCTC-3′.

RELT-Fwd: 5′-TGTGCAACCTCCTCAAGCGGAA-3′.

RELT-Rev: 5′-TGGTGTCCTCATTGGCATCCTC-3′.

RELL-1-Fwd: 5′-GCCCACTAACAAGTCCAGAGAG-3′.

RELL-1-Rev: 5′-CAGACATCAGGCTTCTCGGTTC-3′.

RELL-2-Fwd: 5′-GCTCCAAGGAAGGAAAAAGCCG-3′.

RELL-2-Rev: 5′-TCACGGTGTTCGTGCAGTCCAT-3′.

Western blotting, co-immunoprecipitation (co-IP), and subcellular fractionation. Protein lysates of tissue culture cells were prepared for Western blot analysis, as described previously [34]. Endogenous detection of RELTfms in different cell lines via Western blot used the following polyclonal antibodies for RELTfms: RELT-goat (R&D Systems, cat: AF1385), RELL1-rabbit (Sigma-Aldrich, cat: HPA013377), and RELL2-rabbit (Novus NBP3-10023). Mouse antibodies directed against either β-actin (Santa Cruz Biotechnology, cat: sc-8432) or GAPDH (Santa Cruz Biotechnology, sc-47724) were used for loading controls. Western blotting to confirm recombinant expression of proteins was conducted using an anti-DYKDDDDK (equivalent to Flag) antibody (Cell Signaling Technology, cat: 8146) and an anti-HA antibody (Cell Signaling Technology, cat: 3724). Cleaved PARP detection was conducted using an antibody to detect cleaved PARP (Cell Signaling Technology, cat: 5625). To determine interactions between Flag-tagged OXSR1 kinase and different HA-tagged RELT constructs, 293 cells (≈5 × 10^6^) were transfected with 5 µg of the indicated expression plasmids (10 µg total), and co-IP was performed 24 h after transfection using the FLAG immunoprecipitation kit (Sigma-Aldrich, cat: FLAGIPT1-1KT) according to the manufacturer’s instructions. A 30 µL aliquot of protein lysate was saved prior to the Co-IP procedure for Western analysis to verify protein expression. For subcellular fractionation, 231 and 293 cells (≈5 × 10^6^) were transfected with an expression plasmid for HA-RELT using either Transfex (ATCC, cat: ACS-4005) or Lipofectamine 3000, respectively (ThermoFisher Scientific, cat: L300008). Then, 24 h after transfection, cells were harvested, a subcellular protein fractionation kit (ThermoFisher Scientific, cat: 78840) was used according to the manufacturer’s instructions, and results were analyzed using Western blotting. Recombinant RELT was detected with an anti-HA antibody (Cell Signaling Technology, cat: 3724), and endogenous RELT was detected with a polyclonal rabbit antibody (Invitrogen, cat: PA5-21563). Lamin A was detected using a mouse monoclonal antibody (Santa Cruz Biotechnology, cat: sc-56137) to verify the integrity of nuclear versus cytosol fractions. Secondary antibodies goat anti-rabbit IR-800 (LI-COR, cat: 926-32211) and anti-mouse IR-680 (LI-COR, cat: 926-68020) secondary antibodies used for Western blotting were purchased from LI-COR Biosciences. 

Immunohistochemistry (IHC). IHC staining was performed as described previously [35] using a 1:100 overnight incubation with a RELT polyclonal antibody (Invitrogen, cat: PA521563). A negative control was routinely performed in an identical fashion to the other samples, with the exception that the primary RELT antibody was omitted. IHC was performed on 10 cases of patient-matched cancerous versus non-cancerous mammary tissue. For each case, a minimum of 3 sections of cancerous tissue and 3 sections of non-cancerous mammary tissue were stained. Images of sections with the clearest staining were chosen for quantitative analysis. The intensity of the RELT immunostaining was performed as previously described [22] to assign intensity values based on the semi-quantitative scale of 0 to 3, where 0 is no staining, 1 is weak, 2 is moderate, and 3 is strong. Immunostaining intensity scores were calculated by using the following formula: weighted signal intensity = percentage of immunostained cells × average intensity score. A one-way ANOVA test was used to analyze the statistical significance between the different groups.

Cell death luciferase assays. ≈5 × 10^4^ cells were plated in 12-well plates, and the next day, they were transiently transfected with 1.0 µg of the indicated plasmids using Lipofectamine 3000 and assayed for cell death 48 h after transfection utilizing either the CellTiter-Glo^®^ 2.0 ATP assay (Promega Corporation, cat: G9242) or the Caspase-Glo 3/7 assay (Promega Corporation, cat: G8091), according to the manufacturer’s instructions. Empty vector was utilized as a negative control to account for effects induced by transfection of DNA. Addition of 100 mM of ATP (Sigma-Aldrich, cat: A2383-1G) or 0.3 M of hydrogen peroxide (Sigma-Aldrich, cat: H1009) for six hours was used as positive or negative controls, respectively, for the CellTiter-Glo^®^ ATP assay. Luminescence was measured in relative light units (RLU). Transfections for each sample were performed in duplicate, and each transfection reaction was measured in triplicate; each individual data point, therefore, represents an average of six data points for an individual experiment. Each experiment was conducted at least three times, and representative results are shown. Dixon’s Q-Test was used to remove a maximum of one outlier per data set, and a two-tailed Student T-test was used to determine the statistical significance.

Flow cytometry. For the detection of surface RELT expression, single-cell suspensions were prepared, and cells were stained at a concentration of 1 million cells per ml with an anti-hRELT/TNFRSF19L Alexa Fluor^®^ 647 conjugated antibody (R&D, cat: FAB1385R) for 30 min prior to flow cytometry. Flow cytometry was performed using a BD FACSCaliburTM Flow Cytometer. Data were analyzed using BD Biosciences FlowJoTM10 (Ashland, OR, USA). For cell death experiments, 4.0 × 10^5^ cells were plated in 6-well plates with DMEM medium containing 2% FBS. Cells were transfected the next day with 2.5 µg of the indicated constructs using Transfex transfection reagent according to the manufacturer’s instructions. Each experiment also utilized a group of untreated cells to assess baseline cell viability and serve as a negative control for each treated condition. The cells were prepared for flow cytometry using annexin V (FisherScientific, cat: 50-403-828) and propidium iodide (FisherScientific, cat: BDB556463) stains 48 h after transfection according to the manufacturer’s recommendations. For experiments examining Caspase 3/7 activation, cells were stained with Caspase-3/7 and SYTOX 48 h after transfection according to the manufacturer’s instructions (Invitrogen, cat: C10427). Raw data from experiments were analyzed using FlowJo software and converted to dot plots to compare cell death between groups. For treatment of cells with doxorubicin (Sigma-Aldrich, cat: D1515) or paclitaxel (Sigma-Aldrich, cat: T7191), the media were replaced with fresh DMEM medium (2% FBS) containing either doxorubicin or paclitaxel agents diluted into DMSO at the indicated concentrations 18 h after transfection. Flow cytometry was performed 24 h after treatment with the indicated agents, as described above. 

Xgal morphology assays. ≈1 × 10^5^ cells were plated in 6-well plates and transfected with 1.0 µg of the indicated expression plasmids in addition to 0.2 µg of an expression plasmid for β-galactosidase using Lipofectamine 3000. X-gal morphology assay was performed 48 h after transfection, as described previously [10]. Briefly, a minimum of 6 total viewing fields were examined under 20× magnification, and the percentage of blue cells that exhibited apoptotic morphology (rounded) was quantified. Experiments were performed a minimum of three times, a Dixon’s Q-Test was used to remove a maximum of one outlier per data set, and a two-tailed Student T-test was used to determine the statistical significance.

Immunofluorescence (IF). 231 cells (≈1 × 10^5^) were plated on sterile coverslips in six-well plates and transfected with the indicated expression plasmids using Transfex. IF was performed 24 h after transfection, as described previously [34]. The anti-HA antibody (Cell Signaling Technology, cat: 3724) was utilized to detect recombinant RELT, and polyclonal antibodies were utilized to detect endogenous RELT (Invitrogen, cat: PA521563, and ThermoFisher, cat: PA584568). Anti-rabbit IgG 488 conjugate (Cell Signaling, cat. 4412) secondary antibodies were utilized to visualize RELT-antibody complexes. Vectashield with antifade mounting medium with DAPI (Vector Laboratories, cat: H-2000-10) was added before mounting the coverslips to visualize the nucleus.

## 3. Results

### 3.1. Expression of RELTfms in Different Cancer Cell Lines

**Figure 1:**
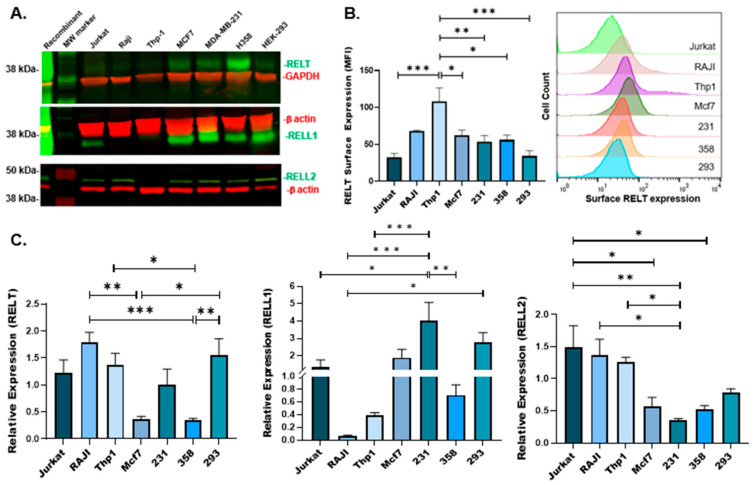
Expression of RELT, RELL1, and RELL2 in human cancer cell lines. (**A**) Western blotting was conducted on protein lysates created from the indicated cell lines: Jurkat (T-cell leukemic) cell line, RAJI (Burkitt’s lymphoma), Thp-1 (Acute Monocytic Leukemia), MCF-7 (breast cancer), MDA-MB-231 (metastatic breast cancer), H358 (lung cancer), and HEK-293 (human kidney embryonic epithelial cell line). A primary polyclonal goat antibody directed against RELT and rabbit polyclonal antibodies directed against RELL1 and RELL2 were used as indicated. Mouse monoclonal antibodies directed against either GAPDH or beta-actin were used as loading controls. Secondary antibodies conjugated to infrared dyes were used to visualize GAPDH and beta-actin (IR 680-red) and RELT family members (IR-800-green). HEK 293 cells transiently transfected with plasmids overexpressing recombinant RELT family members (recombinant) were used as the positive control. Sizes of molecular weight (MW) marker in kilodaltons (kDa) are indicated; the predicted sizes of the proteins based on amino acid sequence alone are as follows: RELT—46 kDa, RELL1—29 kDa, RELL2—32 kDa, β-actin—42 kDa, and GAPDH—36 kDa, respectively. (**B**) Detection of surface RELT expression in human cancer cell lines. An anti-hRELT/TNFRSF19L Alexa Fluor 647 conjugated antibody was used to stain samples created from the indicated cell lines and analyzed using flow cytometry to quantify surface expression of RELT. Mean fluorescence index (MFI) was compared across all 7 cell lines using BD Biosciences FlowJo 10. Comparison of unstained and stained histograms for each cell line are shown in Supplemental Figure S1. (**C**) Expression of RELT, RELL1, and RELL2 mRNA in human cancer cell lines. RNA isolation, cDNA synthesis, and quantitative real time-PCR (RT-PCR) were performed on the indicated cell lines. β-actin was used as the housekeeping gene for normalization. The relative expression of the targets was calculated using 2^−∆∆CT^ method. All experiments shown in Figure 1 show a representative of at least 3 independent experiments. * Indicates *p* ≤ 0.05, ** Indicates *p* ≤ 0.01, *** indicates *p* ≤ 0.001.

We previously published data demonstrating that RELT protein is expressed at high levels in the spleen and lymph nodes and that a truncated version of RELT was prominent in peripheral blood leukocytes [11]. We sought to further explore the expression of RELTfms at the mRNA and protein levels, including RELL1 and RELL2, whose expression at the protein level was not reported previously. Cell lysates from various cancer lines were analyzed using quantitative real-time RT-PCR (qRT-PCR) and Western blotting. Surprisingly, RELT exhibited much stronger protein expression in cell lines representing either breast (MDA-MB-231 and MCF7) or lung (H358) cancer in comparison to cell lines of hematopoietic origin (Figure 1A). An analysis of surface expression using flow cytometry revealed a significantly higher amount of surface RELT expression in monocytes (Thp1) in comparison to other cell lines (Figure 1B). RELT mRNA expression was highest in Thp1, RAJI (B-cells), and HEK-293 (293) cells (Figure 1C), with H358 and MCF7 cells yielding the lowest number of *RELT* transcripts. It was noted that the transcript and protein levels of RELT did not correlate well with each other, especially for H358 and MCF7, in which higher protein levels were associated with lower levels of transcripts. Similarly, RELT protein was detected at lower levels in Jurkat, Raji, Thp1, and 293 cells, yet these cell lines had more pronounced levels of RELT mRNA transcription. It should be noted that RNA molecules transcribed from the RELL1 gene exhibit multiple functions [15,16,17], and long non-coding RELL2 transcripts were observed [18]. Since the genes for the three RELTfms were likely created by duplication and divergenece, it would be expected that they share functions, and it would be reasonable to predict that RNA molecules transcribed off the RELT promoter may also possess independent function, which may help explain the differences between RNA and protein levels shown in Figure 1, in addition to potential mechanisms of post-transcriptional regulation of protein expression.

High amounts of RELL1 protein were expressed in the breast and lung cancer cell lines, although a significant amount of RELL1 protein was also expressed in 293 cells (Figure 1A). Jurkat (T leukemic cell line) exhibited the highest protein expression levels of RELL1 and RELT compared to the other hematopoietic cell lines (Figure 1A). RELL1 mRNA expression was highest in 293 and MDA-MB-231 (231) cell lines, with Thp-1 and RAJI yielding the lowest number of transcripts (Figure 1C). RELL2 migrated higher than the predicted size of 32.4 kDa based on the amino acid sequence alone, yet similar migration of RELL2 was observed previously [9,10], suggesting that RELL2 might be subjected to extensive post-translational modifications. RELL2 exhibited relatively consistent levels of expression across all cell lines, albeit with less protein expressed in Thp1 cells compared to the other cancer cell lines tested. RELL2 mRNA was most readily detected in the Thp-1, RAJI, and Jurkat cell lines, while H358 and 231 revealed the fewest RELL2 transcripts. There were some noted discrepancies between RELL2 RNA and protein, notably with regard to Thp1, in which considerable levels of RNA, yet not protein, were observed. However, long non-coding RELL2 RNA transcripts were identified in intrahepatic cholangiocarcinoma samples [18], and future experiments are required to further elucidate the function of RNA molecules expressed from the genes of RELTfms.

**Figure 2:**
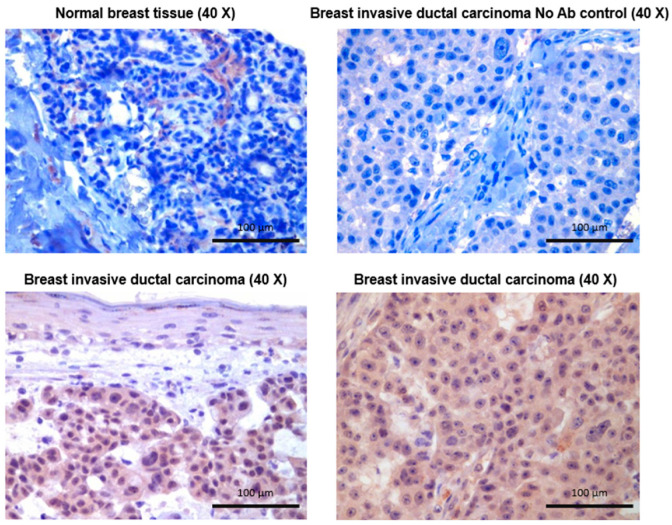
Expression of RELT in human breast cancer biopsies. Immunohistochemistry was performed on both normal mammary tissue and patient-matched breast cancer using an antibody directed against RELT, as described in Materials and Methods. An example of a control is shown in which the RELT primary antibody was omitted; otherwise, the IHC was performed in the exact same manner as the other samples, with the secondary antibody, to verify the specificity of the staining for the RELT antibody. Scale bars of 100 µm are indicated.

Due to the unexpected finding that RELT is expressed at very high levels in cell lines representing BC, we sought to explore the expression of RELT in biopsies of human BC using immunohistochemistry (IHC). Representative examples of the IHC staining observed are shown in Figure 2. RELT staining was observed to be predominantly cellular for normal mammary tissues and was observed to be present in the vicinity of ducts and blood vessels. RELT staining was prominently cytosolic for most cancerous specimens (Figure 2). Six biopsies of benign normal mammary tissue were compared with patient-matched malignant tissue, in addition to four additional malignant biopsies. Quantification of RELT staining intensity demonstrated higher tissue staining intensity in biopsies of malignant BC versus patient-matched benign controls (Table 1).

**Figure 3:**
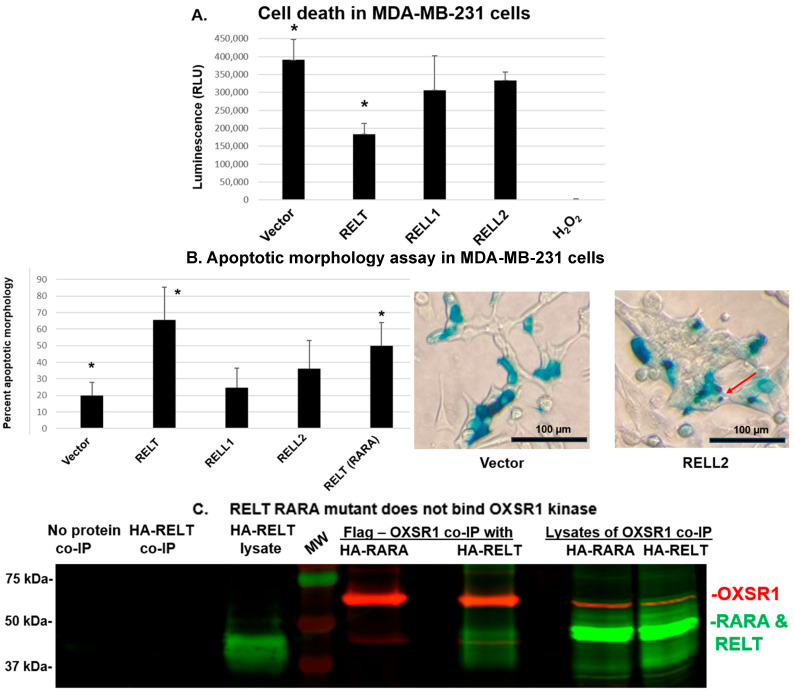
RELT expression induces death in MDA-MB-231 (231) cells. (**A**) ATP luciferase assay. The 231 cells were transfected with the indicated plasmids, and cellular ATP levels were measured 48 h after transfection, as described in Materials and Methods. Hydrogen peroxide treatment was used as a negative control. Treatment of cells with ATP led to an average luciferase value at least one order of magnitude higher than empty vector. Student’s T-test was used to determine significance: * indicates *p* < 0.001 between RELT and vector. A representative of three separate experiments is shown. (**B**) Apoptotic morphology assay. The 231 cells were transfected with the indicated constructs as well as an expression plasmid for β-galactosidase. Cells were stained with X-gal 48 h after transfection, as described in Materials and Methods. Representative images of cells transfected with either empty vector or an expression plasmid for RELL2 are shown at 20× magnification; the appearance of small apoptotic bodies is indicated with a red arrow. Scale bar of 100 µm is indicated. Results are graphically represented as the percentage of β-galactosidase-positive cells that are undergoing cell death based on morphological appearance. A representative of three separate experiments is shown. * Indicates *p* < 0.01 between Vector and RELT and *p* < 0.01 between Vector and RARA. (**C**) RELT RARA mutant does not bind OXSR1 kinase. A RELT mutant construct disrupting the RFRV sequence at positions 349–352 predicted to be required for binding the OXSR1 kinase was created, as described in Materials and Methods. HEK-293 cells were transfected with the indicated expression plasmids, and protein lysates were harvested 24 h after transfection for FLAG co-immunoprecipitation (co-IP) followed by Western blotting, as described in Materials and Methods. Control co-IP experiments were conducted using either control 231 lysates with no overexpressed protein, or co-IP with HA-RELT alone, yet no FLAG-protein, as indicated. Flag-tagged OXSR1 was visualized with a mouse anti-FLAG Ig and subsequent anti-mouse Ig conjugated to IR-680 to visualize OXSR1 as a red band. Rabbit anti-HA Ig and anti-rabbit secondary Ig conjugated with IR-800 enabled visualization of either wild-type RELT or the RARA mutant as green bands. Predicted sizes of proteins based on amino acid sequences alone: OXSR1 kinase—58 kDa and RELT—46 kDa.

Having confirmed that RELT family members are upregulated in cell lines representing BC, we sought to determine whether RELT family members could induce apoptosis in 231 cells, a cell line representative of triple-negative, hormone-independent BC (TNBC), an aggressive and very difficult cancer to treat [36]. The transient overexpression of RELT family members significantly increased the death of 231 cells, as indicated by an ATP luciferase assay (Figure 3A). Similarly, the expression of RELTfms in 231 cells induced apoptotic morphology, as assessed using an X-gal morphology assay (Figure 3B). The expression of RELTfms, especially RELT and RELL2, induced increased cell rounding, indicative of apoptotic cells, similar to what was reported for RELT expression in 293 cells [10]. Additionally, the appearance of small structures that appear to be apoptotic bodies, which are extracellular vesicles released by dying cells [37], could be seen. Additional images of X-gal morphology results are shown in Supplemental Figure S2.

Previous results demonstrated that RELTfms activate the p38 pathway when phosphorylated by the closely related kinases OXSR1 or SPAK [11,12]. A mutant of RELT was constructed in which the conserved RFRV binding site for the OXSR1 and SPAK kinases at amino acid positions 349–352 [38] was changed to RARA, as described in the Materials and Methods. The RFRV → RARA mutation was confirmed by DNA sequencing. Co-immunoprecipitation experiments demonstrated that wild-type RELT, yet not the RARA mutant, physically interacts with the OXSR1 kinase (Figure 3C). The transfection of cells with the RARA mutant of RELT also significantly increased the induction of apoptotic morphology in 231 cells, indicating that phosphorylation by the OXSR1 and SPAK kinases is not required for RELT to induce apoptotic morphology (Figure 3B).

**Figure 4:**
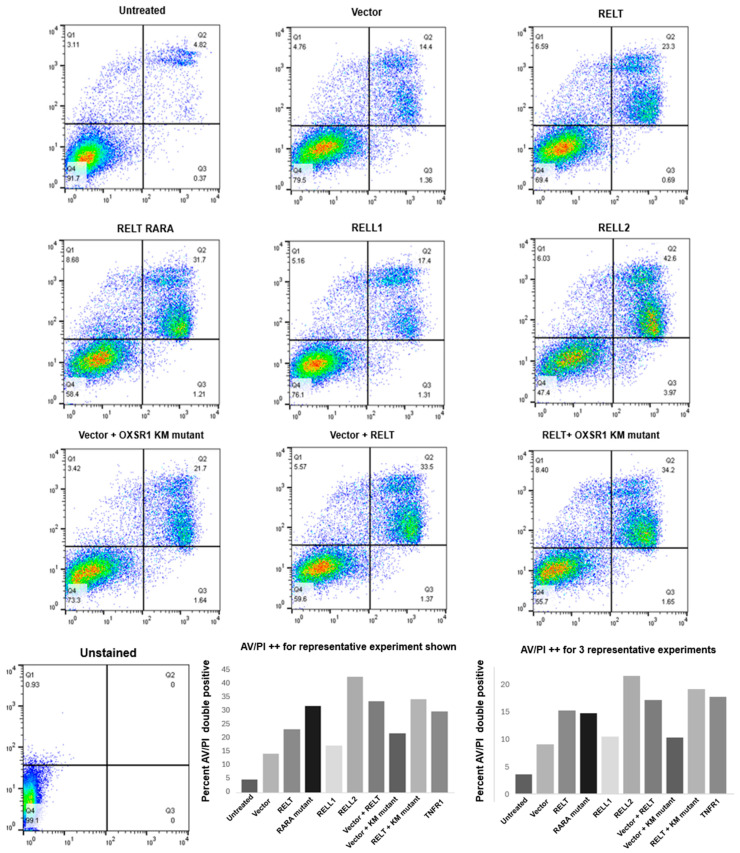
RELT expression induces phosphatidylserine externalization in MDA-MB-231 (231) cells. The 231 cells were transfected with the indicated plasmids, and flow cytometry was conducted 48 h after transfection to determine phosphatidylserine externalization by annexin V (AV) staining and cell death through propidium iodide (PI) staining, as described in Materials and Methods. Cells were separated based on their staining for AV (X-axis) and PI (Y-axis), and the results were quantified based on double-positive AV/PI staining. A representative of three separate experiments is shown. Quantification of the results (**left**) and quantification of three representative experiments (**right**) are shown.

We sought to further investigate the mechanism of RELT-induced death in 231 cells. RELTfms, including the RARA mutant, induced death in 231 cells via an apoptotic pathway, as assessed using annexin V (AV) and propidium iodide (PI) flow cytometry staining (Figure 4). These results indicate that RELT overexpression induces phosphatidylserine (PS) externalization, an early characteristic step of apoptosis that promotes the uptake of apoptotic cells by macrophages [39]. Similarly, RELL1 and RELL2 also induced apoptosis more than the vector control. The magnitude of apoptosis induced by RELL2 was similar to or greater than RELT in different experimental trials, yet RELL1 consistently induced less apoptosis than RELT or RELL2, as measured via AV/PI staining in all experiments conducted. The RELT RARA mutant was capable of inducing apoptosis to a similar extent as RELT. Furthermore, the overexpression of a kinase-dead K46M mutant of OXSR1 that we previously demonstrated had a diminished ability to phosphorylate RELT [9] did not disrupt the ability of RELT to induce apoptosis (Figure 4). We previously reported the ability of RELTfms to induce death in epithelial cells using HEK-293 (293) cells as a model cell line [10], and therefore, we also used flow cytometry to test the ability of RELTfms to induce death in 293 cells. RELTfms also induced death in 293 cells, with RELL1 consistently inducing less apoptosis than RELT or RELL2 (Supplemental Figure S3). Like 231 cells, the ability of RELT to induce apoptosis was not abrogated by either the presence of the RELT RARA mutation or the OXSR1 K46M mutation in 293 cells, providing additional evidence that the death pathway induced by RELT does not require phosphorylation by OXSR1.

**Figure 5:**
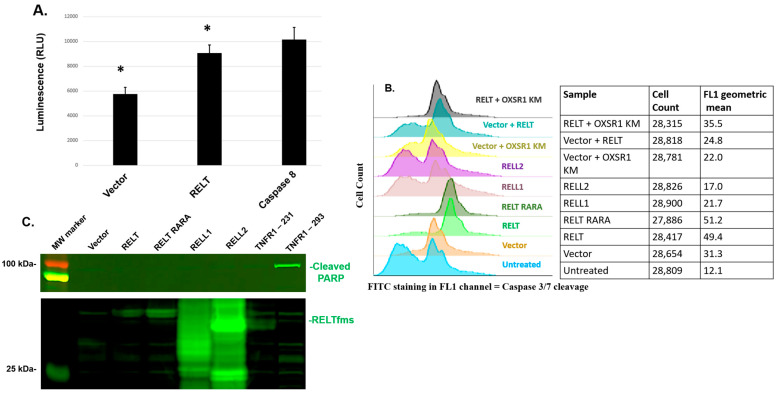
RELT activates cleavage of Caspase-3/7 but not PARP in MDA-MB-231 (231) cells. (**A**) Measurement of Caspase 3/7 activity using luciferase assay. The 231 cells were transfected with the indicated plasmids, and the amount of Caspase 3/7 cleavage was measured 48 h after transfection using a luciferase assay designed to detect Caspase 3/7 cleavage, as described in Materials and Methods. A Student’s T-test was used to determine significance: * indicates *p* < 0.05 between RELT and vector. (**B**) Measurement of Caspase 3/7 activity by flow cytometry. The 231 cells were transfected with the indicated plasmids, and Caspase 3 activity was measured via flow cytometry 48 h after transfection, as described in Materials and Methods. (**C**) Testing whether PARP is cleaved by RELT, RELL1, and RELL2 in MDA-MB-231 cells. The 231 cells were transfected with the indicated constructs, and Western blotting was performed 48 h after transfection, as described in Materials and Methods. A primary rabbit antibody (Ig) directed against cleaved PARP and an anti-rabbit IR 800 conjugated Ig were utilized to visualize cleaved PARP as green bands. An anti-HA antibody was used to verify the expression of recombinant RELT, RELL1, and RELL2 in 231 cells. Representatives of at least 3 experiments are shown for all results in Figure 5.

A luciferase assay specific for caspases 3 and 7 demonstrated increased Caspase 3/7 activity in 231 cells transfected with RELT versus vector control (Figure 5A). The initiator caspase, Caspase 8, served as a positive control, as it directly cleaves and activates Caspase 3 [40,41]. Flow cytometry analysis confirmed that RELTfms activate Caspases 3/7 and that phosphorylation of RELT by OXSR1 was not required for Caspase 3/7 activation, as the expression of either the RELT RARA mutation or the kinase-dead OXSR1 K46M mutant did not impede the ability of RELT to activate Caspase 3/7 (Figure 5B). Curiously, neither RELL1 nor RELL2 consistently increased Caspase 3/7 activation in comparison to 231 cells transfected with the vector, despite consistently increasing the amount of apoptosis (Figure 4) and apoptotic morphology (Figure 3B) in comparison to the vector. PARP is an abundant protein in cells required for DNA repair in addition to many other activities [42], and the cleavage of PARP by caspases has long been considered a hallmark of apoptosis [43]. We therefore tested whether 231 cells overexpressing RELTfms experienced an increase in PARP cleavage by using Western blotting with a primary antibody specific for the large 89 kDa fragment produced by PARP cleavage, as described in the Materials and Methods section. The overexpression of RELTfms in 231 cells did not enhance the cleavage of PARP despite adequate expression of the recombinant proteins (Figure 5C). The antibody was functioning properly, as overexpression of either TNFR1 or Caspase-8 induced the cleavage of PARP in 293 cells. RELT was also capable of inducing Caspase 3/7 activation in 293 cells (Supplemental Figure S4), and unlike 231 cells, the expression of RELT significantly enhanced the appearance of the cleaved 89 kDa PARP fragment in comparison to the empty vector control (Supplemental Figure S5). Similarly, the RELT RARA mutant also significantly enhanced PARP cleavage versus empty vector plasmid, indicating that binding of the OXSR1 kinase is not required for RELT-induced death in 293 cells. RELL1 enhanced the cleavage of PARP in 293 cells less efficiently than RELT or RELL2. Collectively, these findings enhance our understanding of the mechanism by which RELTfms induce death in 231 and 293 cells.

### 3.2. Ability of RELT to Sensitize MDA-MB-231 (231) Cells to Chemotherapeutic Agents

**Figure 6:**
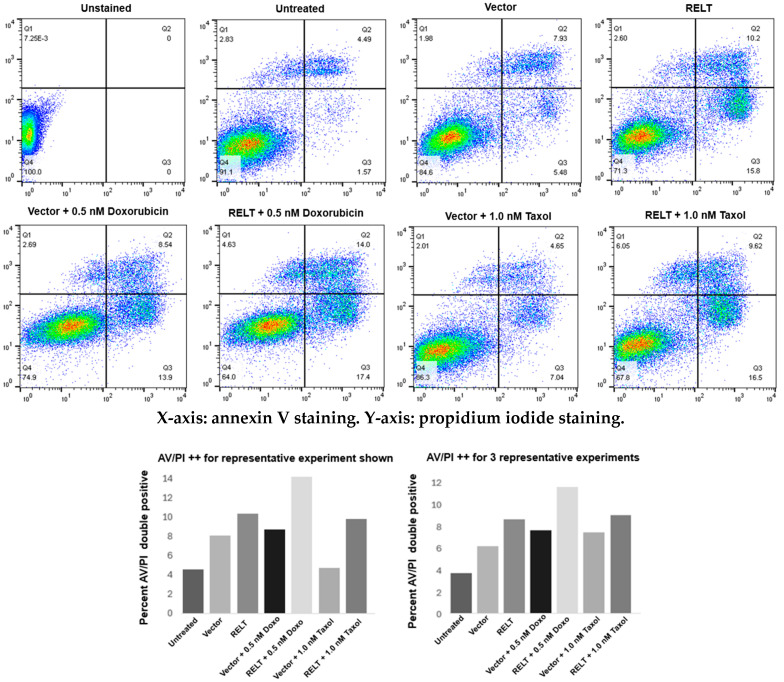
Induction of MDA-MB-231 (231) cell death by chemotherapeutic agents overexpressing RELT. Untransfected cells served as a control for unaltered cells. The 231 cells were transfected with either RELT or empty vector and treated with the indicated chemotherapeutic drugs 24 h after transfection, as described in Materials and Methods. Cells were analyzed 48 h after transfection by annexin V/propidium iodide (AV/PI) staining. Cells were separated based on their staining for AV (X-axis) and PI (Y-axis), and the results were quantified based on double-positive AV/PI staining. A representative of three separate experiments is shown. Quantification of the results (**left**) and quantification of three representative experiments (**right**) are shown.

Given the expression of endogenous RELT in 231 cells and the importance of chemotherapeutic agents to the treatment of BC, we sought to test whether the expression of RELT sensitizes 231 cells to two different chemotherapeutic agents commonly used in the treatment of BC, doxorubicin and paclitaxel (Taxol). Doxorubicin intercalates into DNA and inhibits the ability of Topoisomerase II to relax DNA, which interferes with DNA replication, ultimately leading to apoptosis [44]. The overexpression of RELT enhanced the number of apoptotic 231 cells in response to doxorubicin treatment versus the vector control (Figure 6). Taxol stabilizes microtubules, prevents their disassembly, and blocks attachment of microtubules to the kinetochore, thereby blocking progression through the cell cycle and initiating apoptosis. The overexpression of RELT prior to taxol treatment did not significantly alter the amount of death in 231 cells. These are the first reported results indicating RELT may sensitize BC cells to chemotherapeutic agents. However, RELT also sensitizes BC cells to apoptotic death, and the possibility that the increased death in Figure 6 represents an additive effect of RELT and doxorubicin inducing death by independent pathways can not be excluded.

### 3.3. RELT Localization in MDA-MB-231 (231) Cells

**Figure 7:**
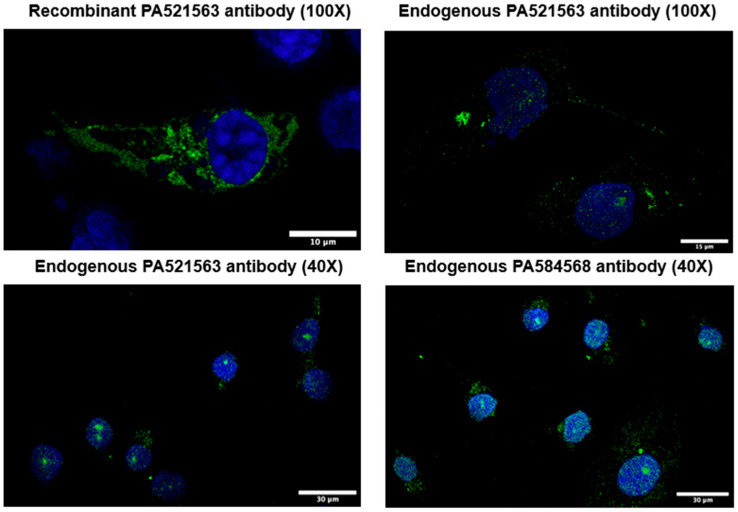
Cellular localization of RELT in MDA-MB-231 (231) cells as assessed via immunofluorescence (IF). Localization of recombinant and endogenous RELT in 231 cells as assessed using IF, as described in Materials and Methods. The primary antibodies used were PA521563, created with RELT residues 35–396 used as an immunogen, and PA584568, created with RELT residues 205–274 used as an immunogen. RELT is visualized as green, and cells were stained with DAPI to visualize the nucleus as blue. Scale bars corresponding to 10, 15, or 30 µm are indicated.

We previously reported that recombinant RELT exhibited punctate cytosolic staining when expressed by itself in either Cos-7 cells [9] or 293 cells [22,34] yet localized at the plasma membrane if co-expressed with RELL1 or RELL2 in Cos-7 cells [9]. We sought to test the expression of RELT in 231 cells using both immunofluorescence (IF) and Western blotting of subcellular fractions. Recombinant RELT in 231s exhibited punctate staining yet was also associated with membrane extensions. The expression of endogenous RELT in 231 cells also exhibited punctate staining in the cytosol, yet unlike recombinant RELT, endogenous RELT in 231 cells exhibited a significant amount of nuclear staining (Figure 7). Similar results were observed when using two separate RELT antibodies from ThermoFisher, including one antibody that used the intracellular domain (ICD) of RELT as the immunogen. We consulted the Human Protein Atlas (HPA) [45] and found that RELT localized to the nucleoplasm in several cell lines representing prostatic adenocarcinoma (PC-3), rhabdomyosarcoma (RH-30), keratinocytes (HaCaT), and osteosarcoma (U-2 OS), although cytosolic staining was also observed in some of the cell lines such as PC-3 https://v23.proteinatlas.org/ENSG00000054967-RELT/subcellular (accessed on 15 November 2024. Localization to the nucleolus was observed, with two separate antibodies from Atlas/Sigma-Aldrich created using different portions of the ICD as the immunogen, 205–274 and 360–425 (Supplemental Figure S6). For reference, the transmembrane domain of RELT is between residues 164–186, and the translated protein contains 430 residues.

**Figure 8:**
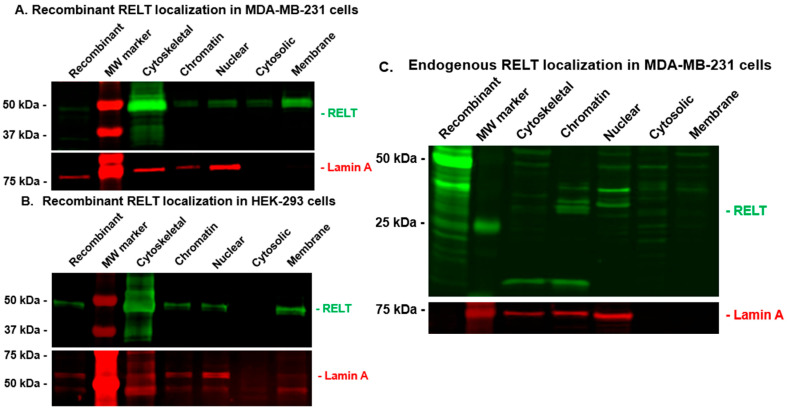
RELT localization in cytoskeleton and chromatin in MDA-MB-231 (231) and HEK-293 cells. Cell fractions were created, and equal amounts of protein were analyzed using Western blotting, as described in Materials and Methods. (**A**,**B**) Recombinant RELT localization in 231 and HEK-293 cells. The indicated cell lines were transiently transfected with an expression plasmid for recombinant HA-tagged RELT, and protein lysates were harvested for fractionation 24 h after transfection. Recombinant RELT was detected using a rabbit anti-HA antibody and secondary anti-rabbit IR 800 antibody to visualize RELT as green bands. (**C**) Endogenous RELT localization in 231 cells. Protein lysates and cell fractions were created from untransfected 231 cells, and endogenous RELT was visualized as green bands with a rabbit polyclonal antibody combined with a secondary anti-rabbit IR 800 antibody. A mouse anti-Lamin A antibody, followed by incubation with an anti-mouse IR 680, was utilized to visualize Lamin A as red bands.

We also used Western blotting of subcellular fractions to analyze the localization of RELT in 231 and 293 cells, as described in Materials and Methods. Recombinant RELT was surprisingly associated abundantly in the cytoskeletal fractions of both 231 and 293 cells (Figure 8A,B). RELT was also expressed in the membrane fraction, as predicted by the transmembrane hydrophobic alpha helix typical of TNFRSF members and the observance of surface RELT expression in 231 cells (Figure 1B). Both RELT and Lamin A were also detected in both the soluble nuclear and chromatin-associated fractions, whereas nuclear Lamin A was not detected in the cytosolic fraction of either cell line, verifying the integrity of the cytosolic versus nuclear fractions. Lamin A was also detected in the cytoskeletal fraction, which is expected, as Lamin A is an intermediate filament and therefore a cytoskeletal protein. The detection of endogenous RELT localization in subcellular fractions gave a surprising result, as the size of endogenous RELT was predominantly less than the expected full-length protein size of 46 kDa based on amino acid sequences alone. RELT was observed in all the fractions, yet it was predominantly approximately 40 kDa in the nuclear and cytosolic fractions and slightly smaller in the chromatin fraction. These results indicate that the size of RELT in different cellular compartments may be altered by proteolytic processing or other post-translational modifications.

## 4. Discussion

Breast cancer (BC) is a disease characterized by significant morbidity and mortality, and many types of BC, such as TNBC, remain impervious to treatment [46]. Furthermore, the incidence of BC in young women is increasing, with the most significant increased incidence occurring in non-Hispanic Black women between the ages of 20 and 39 [47]. This report identifies RELT as a protein that is upregulated in cell lines and biopsies of BC and can also induce death in BC. It adds to the growing examples implicating RELTfms as being important in cancer [4]. Previous reports indicate RELT RNA is upregulated in Ras-transformed mouse mammary epithelial cells that are induced to undergo the epithelial-to-mesenchymal transition [48]. Additionally, autoantibodies (autoAbs) against RELT predicted the presence of BC with a diagnostic accuracy of 71% when comparing the serum of 87 healthy women with 87 women with varying stages of BC [49]. These results indicate that RELT is a tumor-associated antigen in BC with sufficiently altered expression to induce an immune response. AutoAbs associated with cancer have been characterized to bind altered proteins created by missense mutations [50], overexpression [51,52], altered glycosylation [53], or altered pathways of protein degradation [54]. It is unclear how effective autoAbs are at clearing tumors [55], yet their association with tumors has generated great interest in their use as a biomarker for cancer due to their specificity, stability in the blood, and the amplifying effects of the immune system in producing the autoAbs. Furthermore, autoAbs can be detected in the blood in the early stages of the disease, as an examination of HER2 and p53 autoAbs determined that they were present in the serum on average at least 150 days before a subsequent BC diagnosis was made [56]. Future experiments are needed to determine whether the detection of autoAbs against RELT can serve as a diagnostic marker for the early detection of BC. Considering that BC is a heterogenous disease, it would be useful to discern whether RELT autoAbs are more commonly associated with specific types of BC, such as luminal A, luminal B, HER2, or TNBC. The overexpression of RELT in lung cancer was shown to serve as the receptor of nanoparticle delivery of chemotherapeutic agents in a mouse model of lung cancer [21]. Surface expression of RELT was observed at similar levels in cell lines representing lung cancer (H358) and breast cancer (MCF7 and 231) (Figure 1B), and it is therefore worth investigating if upregulated RELT can serve as the receptor for chemotherapy delivery systems in patients with breast cancer.

This work provides the first direct evidence that RELTfms can induce death in BC cells. It should be noted that the transfection efficiency of 231 cells varied between 5 and 20% for different experiments, and therefore, when considering the data presented, please note that the effect of RELTfms on the death of 231 cells is severely hindered by the non-transfected cells, which represent the majority of cells being assayed for cell death by either flow cytometry, luciferase assays, or Western blots. RELL2 was previously identified to suppress the migration and invasion of two separate BC cell lines, 4T1 and 231 cells [57]. RELL2 expression is repressed by microRNA-18a in these BC cell lines. Polyphyllin VI (PP-VI) is a saponin and herbal medicine that suppresses microRNA-18a expression, thereby relieving the repression of RELL2 expression. Although treatment of PP-VI was shown to induce apoptosis in 231 cells, PP-VI and microRNA-18a each likely possess additional targets, and the ability of RELL2 itself to induce apoptosis in 231 cells was not directly tested in this previous study.

We previously reported that RELTfms induce DNA fragmentation and apoptotic morphology in 293 cells [10], yet this work is the first to establish that RELT induces PS externalization, shown for both 231 and 293 cells via annexin V staining, providing further evidence that RELT induces cell death via an apoptotic pathway. RELL1 and RELL2 also induced PS externalization in 293 and 231 cells, yet the observation that RELL1 induces less apoptosis when overexpressed in these two cell lines in comparison to either RELT or RELL2 suggests that the signaling pathways by the three RELTfms differ.

This study provides evidence that RELT-induced apoptosis in BC cells is independent of the OXSR1 kinase, whereas the activation of p38 by RELTfms in 293 cells requires phosphorylation by either of the closely related kinases, OXSR1 and SPAK [11,12]. OXSR1 is expressed endogenously in 293 cells [58] and is predicted to be expressed at moderate levels in 231 cells, courtesy of Human Protein Atlas, https://v23.proteinatlas.org/ENSG00000054967-RELT/cell+line#breast_cancer (accessed on 15 November 2024) [45]. Therefore, although we must consider that endogenous OXSR1 is expressed in the cell lines used in this study, the OXSR1 kinase functions as a dimer [59] that undergoes autophosphorylation during its activation [58]. Therefore, the overexpression of a kinase-dead K46M mutation of OXSR1 would be predicted to have a dominant negative effect and decrease the amount of endogenous OXSR1 kinase available to phosphorylate its substrates, such as RELT, as was observed previously [9]. The ability of the RARA mutant of RELT to induce apoptosis despite lacking the sequence required for binding to either the OXSR1 or SPAK kinases strongly suggests that activation of the p38 pathway by RELT is not required for the death pathway induced by RELT and that a possible bifurcation of pathways exists in the intracellular signal transduction pathways activated by RELT. OXSR1 has been reported to be a poor prognostic indicator for BC [60,61] that can promote the metastasis of BC cells [61]. Future studies are needed to establish the physiological significance of RELT-OXSR1/SPAK in BC cells, including whether these interactions activate the p38 pathway in BC cells.

This report provides the first quantitative evidence that RELT activates the executioner caspases 3 and 7 (C3/7). We previously reported that RELT overexpression in 293 cells induces cleavage of Caspase-3 using immunofluorescence [34], an experiment that requires qualitative interpretation. Although RELT activation of Caspase 3/7 was observed in both 231 and 293 cells, it was surprising that enhanced PARP cleavage was only observed in 293 cells but not 231 cells. However, the expression of genes associated with DNA repair is often dysregulated in BC, and low levels of PARP have been reported in 231 cells compared to other BC cell lines [62]. Similarly, it was surprising to note that RELL1 and RELL2 induced both PS externalization and apoptotic morphology in 231 cells yet did not activate C3/7, indicating that these RELT homologs may activate apoptosis by a distinct pathway from RELT. We previously reported that RELT can induce the appearance of apoptotic morphology in 293 cells by a mechanism that is independent of Caspase-8 or FADD [11], so it appears clear that RELTfms induce apoptosis by a mechanism distinct from the canonical pathway used by other TNFRSR members [40,63], as further evidenced by the lack of conserved death domains in RELTfms present in other TNFRSF members that induce death [3]. It would be of great interest to further elucidate the mechanisms downstream of RELT that govern both apoptotic and inflammatory pathways that have been observed in different settings, as this may provide insight into the conflicting reports of whether RELT facilitates or is protective against tumorigenesis. For example, RELT is a good prognostic indicator for small-cell lung cancer [27], yet conversely, it can promote NF-κB activation in multiple myeloma cells [20] and in ESCC [19] and inhibit Caspase 3 cleavage in ESCC [19]. RELT may be similar to other TNFRSF members that can function in either a pro-apoptotic or pro-inflammatory manner, such as TNFR1 [64] and DR3 [65,66]. RELL2 can inhibit tumor cell migration [57] and induce the death of cancer cells ex vivo [32,33] and in vivo [32], yet it is conversely a poor prognostic indicator for several cancers [30,31]. In contrast, thus far, the few reports studying the relationship of RELL1 protein with cancer indicate it is pro-tumorigenic [28,67], and it is interesting to speculate whether this correlates with the decreased ability of RELL1 to induce death in 231 and 293 cells in comparison to other RELTfms.

This work provides the first evidence that RELT may sensitize cancer cells to chemotherapeutic agents, especially doxorubicin, although it cannot be excluded that the results shown in Figure 6 are solely from an additive effect of the pro-apoptotic effects of RELT and either taxol or doxorubicin. RELL2 was previously shown to sensitize pancreatic ductal adenocarcinoma cells to the nucleoside analog gemcitabine [33], and the ability of RELTfms to sensitize cancer cells to chemotherapy is an avenue that needs further exploration. If RELTfms can serve as a therapeutic target to increase the killing of BC cells, we predict that RELT and RELL2 would serve as better targets due to their increased ability to kill BC cells (Figure 3 and Figure 4) and the previously reported ability of RELL2 to inhibit the migration and invasion of BC cell lines [57]. The saponin PP-VI, derived from the medicinal plant *Paris polyphylla*, upregulates the expression of RELL2 in 231 cells by relieving the inhibitory effects of miR-18a on RELL2 expression, and this report also demonstrated that PP-VI also killed BC cell lines. There have been many reports highlighting the anti-cancer activities of saponins produced by this plant (the Chinese name of the plant is Chonglou) [68], and upregulation of the anti-cancer properties of RELL2 via PP-VI should be further explored as a potential treatment for BC.

Additional experiments are needed to better clarify conditions that can upregulate RELT to determine if RELT can be used as a therapeutic target to increase BC death. However, RELT may function in a pro-tumorigenic mechanism depending on the type of cancer, as RELT functions as an oncogene in ESCC [19] and potentially in other cancers. Additionally, multiple lines of evidence indicate that RELT may promote an immunosuppressive environment in cancer. First, as discussed previously, the prominent phenotype of mice lacking RELT was enhanced T-cell responses, including increased killing of tumor cells by CTLs [6], indicating that RELT suppresses T-cells. Furthermore, bioinformatic analysis examining the relationship of RELT with both prostate and renal cancer also indicates that RELT may promote an immunosuppressive environment that favors the tumor [23,25,26]. Future experiments are needed to discern whether RELT may also function to suppress the immune system in BC. The relationship between RELT and cancer may be another example of a protein that can either promote apoptosis or pro-tumorigenic functions, similar to the ability of TNFα treatment to induce either apoptosis or promote tumor growth in TNBC [66].

Kaplan–Meier survival analyses, courtesy of the Human Protein Atlas, https://v23.proteinatlas.org/ENSG00000054967-RELT/pathology/breast+cancer (accessed on 15 November 2024) [45], indicate that high RELT expression may be slightly protective for BC (Supplemental Figure S7), yet the difference was not considered significant. A Kaplan–Meier Plotter database was also used to analyze the association between RELT expression and BC patient survival. The analysis indicates that the longer survival in patients expressing higher levels of RELT is not statistically significant (Supplemental Figure S8). Further research could help clarify RELT’s role in breast cancer, specifically whether it functions in a tumor-promoting or tumor-suppressing capacity across different BC subtypes.

Thus far, reports of RELL1 indicate that it is an oncogene for glioma [28] and a poor prognostic indicator for glioblastoma [67] and colorectal cancer [29]. RELL1 being more consistently associated with a pro-tumorigenic function is consistent with our observations that it killed BC cells less effectively than RELT or RELL2 (Figure 3 and Figure 4). IFNγ downregulates RELL1 expression in a murine macrophage cell line [13], and therefore, the inhibition of pro-tumorigenic functions of RELL1 by IFNγ is another avenue worth exploring.

A surprising observation was that RELT can translocate to the nucleus and interact with chromatin (Figure 7 and Figure 8), consistent with observations available, courtesy of the Human Protein Atlas [45]. Endogenous nuclear localization using immunofluorescence was observed with two distinct antibodies raised with epitopes mapping to the ICD of RELT, with one of the epitopes (205–274) mapping close to the transmembrane domain (164–186) (Figure 7 and Figure S4) and the other epitope (360–425) mapping close to the extreme carboxy terminus of the protein (Supplemental Figure S4). Westerns of subcellular fractionations confirm that RELT migrates to the nucleus and exists in the cytosol in a non-membraneous compartment (Figure 8), indicating that a portion of the ICD is cleaved to produce a water-soluble fragment. Alternatively, RELT could conceivably adopt an alternative conformation in which the predicted transmembrane alpha helix is buried at the center of a globular protein, as has been shown for other proteins previously [69]. It is interesting to note that recombinant nuclear RELT was full-length, whereas endogenous nuclear RELT was smaller than full-length recombinant RELT (Figure 8C), supporting a hypothesis that nuclear RELT represents a truncated ICD fragment of RELT.

This work adds to the growing number of TNFRSF members that have been recently discovered to migrate to the nucleus. The TNFRSF member CD40 translocates to the nucleus, where it functions as a transcription factor [70]. DR5 (TRAIL-R2) is imported into the nucleus through Importin β1 [71] and interferes with the processing of miRNA once in the nucleus [72]. It is interesting to speculate whether the differing effects of RELT on apoptosis [10,11] or NF-κB activation [19,20] could, in part, represent differing functions of RELT based on location—for example, secreted RELT was associated with NF-κB activation in one study [20]. Similarly, membrane-associated DR5 is well established as a protein that effectively kills cancer cells, yet nuclear DR5 promotes the malignancy of tumors [73]. Likewise, the localization of TNFRSF death receptors to either the cytosol or nucleus can promote the resistance of BC to death receptor-targeted therapies [74]. It is interesting to note that the RELT-binding proteins Phospholipid Scramblase 1 (PLSCR1) [34] and MyoD Family Inhibitor domain Containing (MDFIC) [22] also have nuclear functions, as nuclear PLSCR1 activates STAT1 signaling [75] and nuclear MDFIC (also known as HIC) can serve to bind Cyclin T1 and modulate transcription [76] and also sequester transcription factors in the nucleolus [77]. Future experiments are needed to characterize which nuclear components RELT interacts with, whether RELT binds DNA or can serve as a transcription factor, and how potential nuclear functions of RELT relate to cancers such as BC [45].

It is important to emphasize that RELT was also observed in the membrane fraction as expected (Figure 7) and that we confirmed RELT expression on the surface of several cell lines (Figure 1B). Surface expression of RELT was previously detected in mouse lung tumors [21], and surface expression of RELT is required to be cleaved by the ADAM10 protease to produce a secreted diffusible extracellular domain (ECD) signal that is required for enamel development [78]. Additional research is needed to determine whether the ECD produced by ADAM10 cleavage serves other functions, as the expression of both RELT and ADAM10 is predicted to occur in several other tissues besides ameloblasts https://v23.proteinatlas.org/ENSG00000054967-RELT/tissue+cell+type (accessed on 15 November 2024), courtesy of the Human Protein Atlas [45], including ductal cells, which may be of relevance to BC. The cytosolic localization of RELT also needs to be further clarified. Recombinant RELT exhibited a much higher cytosolic/nuclear ratio than endogenous RELT (Figure 7) and was predominantly associated with the cytoskeleton (Figure 8). Future experiments are needed to determine where in the cytosol RELT localizes to, with respect to both non-membraneous compartments, as well as vesicles or membranes associated with the endosomal pathway or the trans-Golgi network, for example, and how these cellular localizations correlate with RELT function.

Lastly, a glaring piece of information lacking is the identification of the ligand for RELT. RELT contains conserved cysteine-rich domains in its ECD that predict binding to a TNFSF ligand [5]. Previous attempts to identify a TNFSF ligand for RELT were unsuccessful [79], and the ligand(s) for both membrane-associated RELT and secreted RELT need to be further elucidated. We postulate that overexpressing RELT in 231 cell lines induces trimerization, as has been shown for other TNFRSF members [80], to mimic the presence of the unidentified TNFSF ligand. RELL1 and RELL2 have been shown to physically bind RELT and have been proposed to serve as adaptor proteins that modulate RELT signaling in the presence of an unknown ligand [9], yet the presence of either RELT homotrimers or RELT heterotrimers containing RELL1 or RELL2 has not been demonstrated. The confirmation of RELT homotrimers and heterotrimers and how their activities differ in BC needs to be elucidated. If RELT does trimerize in response to an unidentified TNFSF trimeric ligand, then it is reasonable to predict that RELT heterotrimers would consist of two molecules of RELT complexed with either RELL1 or RELL2, as the ECDs of RELL1 and RELL2 are truncated and lack the Cys-containing regions used to bind trimeric TNFSF members [3]. However, it is also possible that RELTfms function as dimers or as monomers, as the TNFRSF member p75 (TNFRSF16) functions as a monomer when it binds its ligand neutrophin, a molecule with no resemblance to TNFSF members [81]. It is evident that more research is needed to be conducted on RELT, RELL1, and RELL2, as our knowledge of these evolutionarily conserved proteins is sorely lacking in comparison to other TNFRSF members. Collectively, these results further characterize the RELT with BC and add to a growing list of TNFRSF members implicated in BC [82,83] deserving of further study.

## Figures and Tables

**Table 1:** Quantification of RELT staining in benign versus malignant breast cancer biopsies. RELT protein expression was semi-quantitatively measured in biopsies taken from benign mammary versus malignant tissue.

Sample	RELT Staining Intensity (Mean ± SEM)
Normal (N = 6)	44.2 ± 17.2
Malignant (N = 10)	109.5 ± 16.6

## Data Availability

Data are contained within the article and supplementary materials.

## Supplementary Materials

The following supporting information can be downloaded at: https://www.mdpi.com/article/10.3390/biomedicines12122667/s1, Figure S1: Comparison of unstained and stained flow cytometry histograms used to determine surface RELT expression. Figure S2: X-gal morphology of 231 cells expressing RELT, RELL1, RELL2, and RARA mutant of RELT. Figure S3: RELT family members induce phosphatidylserine externalization in HEK-293 cells. Figure S4: RELT family members induce Caspase 3/7 activation in HEK-293 cells. Figure S5: RELT family members induce the cleavage of PARP by RELT in HEK-293 cells. Figure S6: The cellular localization of endogenous RELT, as assessed by immunofluorescence, courtesy of the Human Protein Atlas. Figure S7: A Kaplan–Meier survival scatter plot of RELT and breast cancer, courtesy of the Human Protein Atlas. Figure S8: Kaplan-Meier survival analysis of breast cancer patients stratified by RELT expression levels.

## References

[1] Aggarwal B.B., Gupta S.C., Kim J.H. (2012). Historical perspectives on tumor necrosis factor and its superfamily: 25 years later, a golden journey. Blood.

[2] Collette Y., Gilles A., Pontarotti P., Olive D. (2003). A co-evolution perspective of the TNFSF and TNFRSF families in the immune system. Trends Immunol..

[3] Hehlgans T., Pfeffer K. (2005). The intriguing biology of the tumour necrosis factor/tumour necrosis factor receptor superfamily: Players, rules and the games. Immunology.

[4] Cusick J.K., Alcaide J., Shi Y. (2023). The RELT Family of Proteins: An Increasing Awareness of Their Importance for Cancer, the Immune System, and Development. Biomedicines.

[5] Sica G.L., Zhu G., Tamada K., Liu D., Ni J., Chen L. (2001). RELT, a new member of the tumor necrosis factor receptor superfamily, is selectively expressed in hematopoietic tissues and activates transcription factor NF-kappaB. Blood.

[6] Choi B.K., Kim S.H., Kim Y.H., Lee D.G., Oh H.S., Han C., Kim Y.I., Jeon Y., Lee H., Kwon B.S. (2018). RELT negatively regulates the early phase of the T-cell response in mice. Eur. J. Immunol..

[7] Kim J.W., Zhang H., Seymen F., Koruyucu M., Hu Y., Kang J., Kim Y.J., Ikeda A., Kasimoglu Y., Bayram M. (2019). Mutations in RELT cause autosomal recessive amelogenesis imperfecta. Clin. Genet..

[8] Nikolopoulos G., Smith C.E.L., Brookes S.J., El-Asrag M.E., Brown C.J., Patel A., Murillo G., O’Connell M.J., Inglehearn C.F., Mighell A.J. (2020). New missense variants in RELT causing hypomineralised amelogenesis imperfecta. Clin. Genet..

[9] Cusick J.K., Xu L.G., Bin L.H., Han K.J., Shu H.B. (2006). Identification of RELT homologues that associate with RELT and are phosphorylated by OSR1. Biochem. Biophys. Res. Commun..

[10] Cusick J.K., Mustian A., Goldberg K., Reyland M.E. (2010). RELT induces cellular death in HEK 293 epithelial cells. Cell Immunol..

[11] Moua P., Checketts M., Xu L.G., Shu H.B., Reyland M.E., Cusick J.K. (2017). RELT family members activate p38 and induce apoptosis by a mechanism distinct from TNFR1. Biochem. Biophys. Res. Commun..

[12] Polek T.C., Talpaz M., Spivak-Kroizman T. (2006). The TNF receptor, RELT, binds SPAK and uses it to mediate p38 and JNK activation. Biochem. Biophys. Res. Commun..

[13] Feng L., Hu J., Zhang W., Dong Y., Xiong S., Dong C. (2020). RELL1 inhibits autophagy pathway and regulates Mycobacterium tuberculosis survival in macrophages. Tuberculosis.

[14] Groza T., Gomez F.L., Mashhadi H.H., Munoz-Fuentes V., Gunes O., Wilson R., Cacheiro P., Frost A., Keskivali-Bond P., Vardal B. (2023). The International Mouse Phenotyping Consortium: Comprehensive knockout phenotyping underpinning the study of human disease. Nucleic Acids Res..

[15] Huang H.S., Huang X.Y., Yu H.Z., Xue Y., Zhu P.L. (2020). Circular RNA circ-RELL1 regulates inflammatory response by miR-6873-3p/MyD88/NF-kappaB axis in endothelial cells. Biochem. Biophys. Res. Commun..

[16] Ding H., Chen H., Dou L., Li Y. (2024). CircRELL1 promotes osteoarthritis progression by regulating miR-200c-3p. Heliyon.

[17] Sang H., Zhang W., Peng L., Wei S., Zhu X., Huang K., Yang J., Chen M., Dang Y., Zhang G. (2022). Exosomal circRELL1 serves as a miR-637 sponge to modulate gastric cancer progression via regulating autophagy activation. Cell Death Dis..

[18] Angenard G., Merdrignac A., Louis C., Edeline J., Coulouarn C. (2019). Expression of long non-coding RNA ANRIL predicts a poor prognosis in intrahepatic cholangiocarcinoma. Dig. Liver Dis..

[19] Yao W., Chen Q., Li S., Jia X., Xu L., Wei L. (2021). RELT promotes the growth of esophageal squamous cell carcinoma by activating the NF-kappaB pathway. Cell Cycle.

[20] Wang H., Wang L., Luan H., Xiao J., Zhao Z., Yu P., Deng M., Liu Y., Ji S., Ma J. (2024). LILRB4 on multiple myeloma cells promotes bone lesion by p-SHP2/NF-kappaB/RELT signal pathway. J. Exp. Clin. Cancer Res..

[21] Jung H., Park S., Gunassekaran G.R., Jeon M., Cho Y.E., Baek M.C., Park J.Y., Shim G., Oh Y.K., Kim I.S. (2019). A Peptide Probe Enables Photoacoustic-Guided Imaging and Drug Delivery to Lung Tumors in K-ras(LA2) Mutant Mice. Cancer Res..

[22] Cusick J.K., Alhomsy Y., Wong S., Talbott G., Uversky V.N., Hart C., Hejazi N., Jacobs A.T., Shi Y. (2020). RELT stains prominently in B-cell lymphomas and binds the hematopoietic transcription factor MDFIC. Biochem. Biophys. Rep..

[23] Cui Y., Shen T., Xu F., Zhang J., Wang Y., Wu J., Bu H., Fu D., Fang B., Lv H. (2022). KCNN4 may weaken anti-tumor immune response via raising Tregs and diminishing resting mast cells in clear cell renal cell carcinoma. Cancer Cell Int..

[24] Guo Y., Pan W.K., Wang Z.W., Su W.H., Xu K., Jia H., Chen J. (2021). Identification of Novel Biomarkers for Predicting Prognosis and Immunotherapy Response in Head and Neck Squamous Cell Carcinoma Based on ceRNA Network and Immune Infiltration Analysis. BioMed Res. Int..

[25] Ge S., Hua X., Chen J., Xiao H., Zhang L., Zhou J., Liang C., Tai S. (2021). Identification of a Costimulatory Molecule-Related Signature for Predicting Prognostic Risk in Prostate Cancer. Front. Genet..

[26] Mei W., Dong Y., Gu Y., Kapoor A., Lin X., Su Y., Vega Neira S., Tang D. (2023). IQGAP3 is relevant to prostate cancer: A detailed presentation of potential pathomechanisms. J. Adv. Res..

[27] Zhang Z., Wu P., Zhang C., Luo Y., Zhang G., Zeng Q., Wang L., Yang Z., Sun N., He J. (2021). Tumor Necrosis Factor Family Member Profile Predicts Prognosis and Adjuvant Chemotherapy Benefit for Patients with Small-Cell Lung Cancer. Front. Immunol..

[28] Jin X., Xie H., Liu X., Shen Q., Wang Z., Hao H., Gu Y. (2020). RELL1, a novel oncogene, accelerates tumor progression and regulates immune infiltrates in glioma. Int. Immunopharmacol..

[29] Kadkhoda S., Darbeheshti F., Rezaei N., Azizi-Tabesh G., Zolfaghari F., Tavakolibazaz S., Taslimi R., Tavakkoly-Bazzaz J. (2021). Investigation of circRNA-miRNA-mRNA network in colorectal cancer using an integrative bioinformatics approach. Gastroenterol. Hepatol. Bed Bench..

[30] Musha K., Ge X., Ablikim N., Lu B., Chen C., Huang J. (2022). Comprehensive Analysis of RELL2 as a Potential Biomarker Associated with Tumor Immune Infiltrating Cells in a Pan-Cancer Analysis. Dis. Markers.

[31] Zhao Y., Niu L.T., Hu L.J., Lv M. (2022). Comprehensive analysis of ECHDC3 as a potential biomarker and therapeutic target for acute myeloid leukemia: Bioinformatic analysis and experimental verification. Front. Oncol..

[32] Tang S.J., Shen H., An O., Hong H., Li J., Song Y., Han J., Tay D.J.T., Ng V.H.E., Bellido Molias F. (2020). Cis- and trans-regulations of pre-mRNA splicing by RNA editing enzymes influence cancer development. Nat. Commun..

[33] Li Z., Qin C., Zhao B., Wang Y., Li T., Zhao Y., Wang W. (2023). DHX38 restricts chemoresistance by regulating the alternative pre-mRNA splicing of RELL2 in pancreatic ductal adenocarcinoma. PLoS Genet..

[34] Cusick J.K., Mustian A., Jacobs A.T., Reyland M.E. (2012). Identification of PLSCR1 as a protein that interacts with RELT family members. Mol. Cell. Biochem..

[35] Maines L.W., Fitzpatrick L.R., French K.J., Zhuang Y., Xia Z., Keller S.N., Upson J.J., Smith C.D. (2008). Suppression of ulcerative colitis in mice by orally available inhibitors of sphingosine kinase. Dig. Dis. Sci..

[36] Chavez K.J., Garimella S.V., Lipkowitz S. (2010). Triple negative breast cancer cell lines: One tool in the search for better treatment of triple negative breast cancer. Breast Dis..

[37] Yu L., Zhu G., Zhang Z., Yu Y., Zeng L., Xu Z., Weng J., Xia J., Li J., Pathak J.L. (2023). Apoptotic bodies: Bioactive treasure left behind by the dying cells with robust diagnostic and therapeutic application potentials. J. Nanobiotechnol..

[38] Delpire E., Gagnon K.B. (2007). Genome-wide analysis of SPAK/OSR1 binding motifs. Physiol. Genom..

[39] Fadok V.A., de Cathelineau A., Daleke D.L., Henson P.M., Bratton D.L. (2001). Loss of phospholipid asymmetry and surface exposure of phosphatidylserine is required for phagocytosis of apoptotic cells by macrophages and fibroblasts. J. Biol. Chem..

[40] Yanumula A., Cusick J.K. (2024). Biochemistry, Extrinsic Pathway of Apoptosis.

[41] Chang H.Y., Yang X. (2000). Proteases for cell suicide: Functions and regulation of caspases. Microbiol. Mol. Biol. Rev..

[42] Li W.H., Wang F., Song G.Y., Yu Q.H., Du R.P., Xu P. (2023). PARP-1: A critical regulator in radioprotection and radiotherapy-mechanisms, challenges, and therapeutic opportunities. Front. Pharmacol..

[43] Kaufmann S.H., Desnoyers S., Ottaviano Y., Davidson N.E., Poirier G.G. (1993). Specific proteolytic cleavage of poly(ADP-ribose) polymerase: An early marker of chemotherapy-induced apoptosis. Cancer Res..

[44] Thorn C.F., Oshiro C., Marsh S., Hernandez-Boussard T., McLeod H., Klein T.E., Altman R.B. (2011). Doxorubicin pathways: Pharmacodynamics and adverse effects. Pharmacogenet. Genom..

[45] Uhlen M., Fagerberg L., Hallstrom B.M., Lindskog C., Oksvold P., Mardinoglu A., Sivertsson A., Kampf C., Sjostedt E., Asplund A. (2015). Tissue-based map of the human proteome. Science.

[46] Gupta G.K., Collier A.L., Lee D., Hoefer R.A., Zheleva V., Siewertsz van Reesema L.L., Tang-Tan A.M., Guye M.L., Chang D.Z., Winston J.S. (2020). Perspectives on Triple-Negative Breast Cancer: Current Treatment Strategies, Unmet Needs, and Potential Targets for Future Therapies. Cancers.

[47] Xu S., Murtagh S., Han Y., Wan F., Toriola A.T. (2024). Breast Cancer Incidence Among US Women Aged 20 to 49 Years by Race, Stage, and Hormone Receptor Status. JAMA Netw. Open.

[48] Johansson J., Tabor V., Wikell A., Jalkanen S., Fuxe J. (2015). TGF-β1-Induced Epithelial-Mesenchymal Transition Promotes Monocyte/Macrophage Properties in Breast Cancer Cells. Front. Oncol..

[49] Zhong L., Ge K., Zu J.C., Zhao L.H., Shen W.K., Wang J.F., Zhang X.G., Gao X., Hu W., Yen Y. (2008). Autoantibodies as potential biomarkers for breast cancer. Breast Cancer Res..

[50] Soussi T. (2000). p53 Antibodies in the sera of patients with various types of cancer: A review. Cancer Res..

[51] Chen Y.T., Scanlan M.J., Sahin U., Tureci O., Gure A.O., Tsang S., Williamson B., Stockert E., Pfreundschuh M., Old L.J. (1997). A testicular antigen aberrantly expressed in human cancers detected by autologous antibody screening. Proc. Natl. Acad. Sci. USA.

[52] Goodell V., Waisman J., Salazar L.G., de la Rosa C., Link J., Coveler A.L., Childs J.S., Fintak P.A., Higgins D.M., Disis M.L. (2008). Level of HER-2/neu protein expression in breast cancer may affect the development of endogenous HER-2/neu-specific immunity. Mol. Cancer Ther..

[53] Wandall H.H., Blixt O., Tarp M.A., Pedersen J.W., Bennett E.P., Mandel U., Ragupathi G., Livingston P.O., Hollingsworth M.A., Taylor-Papadimitriou J. (2010). Cancer biomarkers defined by autoantibody signatures to aberrant O-glycopeptide epitopes. Cancer Res..

[54] Ulanet D.B., Torbenson M., Dang C.V., Casciola-Rosen L., Rosen A. (2003). Unique conformation of cancer autoantigen B23 in hepatoma: A mechanism for specificity in the autoimmune response. Proc. Natl. Acad. Sci. USA.

[55] Kobold S., Lutkens T., Cao Y., Bokemeyer C., Atanackovic D. (2010). Autoantibodies against tumor-related antigens: Incidence and biologic significance. Hum. Immunol..

[56] Lu H., Ladd J., Feng Z., Wu M., Goodell V., Pitteri S.J., Li C.I., Prentice R., Hanash S.M., Disis M.L. (2012). Evaluation of known oncoantibodies, HER2, p53, and cyclin B1, in prediagnostic breast cancer sera. Cancer Prev. Res..

[57] Wang P., Yang Q., Du X., Chen Y., Zhang T. (2019). Targeted regulation of Rell2 by microRNA-18a is implicated in the anti-metastatic effect of polyphyllin VI in breast cancer cells. Eur. J. Pharmacol..

[58] Chen W., Yazicioglu M., Cobb M.H. (2004). Characterization of OSR1, a member of the mammalian Ste20p/germinal center kinase subfamily. J. Biol. Chem..

[59] Lee S.J., Cobb M.H., Goldsmith E.J. (2009). Crystal structure of domain-swapped STE20 OSR1 kinase domain. Protein Sci..

[60] Li Y., Qin J., Wu J., Dai X., Xu J. (2020). High expression of OSR1 as a predictive biomarker for poor prognosis and lymph node metastasis in breast cancer. Breast Cancer Res. Treat..

[61] Li Y., Li L., Qin J., Wu J., Dai X., Xu J. (2021). OSR1 phosphorylates the Smad2/3 linker region and induces TGF-β1 autocrine to promote EMT and metastasis in breast cancer. Oncogene.

[62] Lee K.J., Piett C.G., Andrews J.F., Mann E., Nagel Z.D., Gassman N.R. (2019). Defective base excision repair in the response to DNA damaging agents in triple negative breast cancer. PLoS ONE.

[63] Wilson N.S., Dixit V., Ashkenazi A. (2009). Death receptor signal transducers: Nodes of coordination in immune signaling networks. Nat. Immunol..

[64] Philip M., Rowley D.A., Schreiber H. (2004). Inflammation as a tumor promoter in cancer induction. Semin. Cancer Biol..

[65] Chinnaiyan A.M., O’Rourke K., Yu G.L., Lyons R.H., Garg M., Duan D.R., Xing L., Gentz R., Ni J., Dixit V.M. (1996). Signal transduction by DR3, a death domain-containing receptor related to TNFR-1 and CD95. Science.

[66] Mercogliano M.F., Bruni S., Elizalde P.V., Schillaci R. (2020). Tumor Necrosis Factor α Blockade: An Opportunity to Tackle Breast Cancer. Front. Oncol..

[67] Rose M., Cardon T., Aboulouard S., Hajjaji N., Kobeissy F., Duhamel M., Fournier I., Salzet M. (2021). Surfaceome Proteomic of Glioblastoma Revealed Potential Targets for Immunotherapy. Front. Immunol..

[68] Li J., Jia J., Zhu W., Chen J., Zheng Q., Li D. (2023). Therapeutic effects on cancer of the active ingredients in rhizoma paridis. Front. Pharmacol..

[69] Bateman A., Finn R.D., Sims P.J., Wiedmer T., Biegert A., Soding J. (2009). Phospholipid scramblases and Tubby-like proteins belong to a new superfamily of membrane tethered transcription factors. Bioinformatics.

[70] Lin-Lee Y.C., Pham L.V., Tamayo A.T., Fu L., Zhou H.J., Yoshimura L.C., Decker G.L., Ford R.J. (2006). Nuclear localization in the biology of the CD40 receptor in normal and neoplastic human B lymphocytes. J. Biol. Chem..

[71] Kojima T., Morikawa Y., Copeland N.G., Gilbert D.J., Jenkins N.A., Senba E., Kitamura T. (2000). TROY, a newly identified member of the tumor necrosis factor receptor superfamily, exhibits a homology with Edar and is expressed in embryonic skin and hair follicles. J. Biol. Chem..

[72] Haselmann V., Kurz A., Bertsch U., Hubner S., Olempska-Muller M., Fritsch J., Hasler R., Pickl A., Fritsche H., Annewanter F. (2014). Nuclear death receptor TRAIL-R2 inhibits maturation of let-7 and promotes proliferation of pancreatic and other tumor cells. Gastroenterology.

[73] Bertsch U., Roder C., Kalthoff H., Trauzold A. (2014). Compartmentalization of TNF-related apoptosis-inducing ligand (TRAIL) death receptor functions: Emerging role of nuclear TRAIL-R2. Cell Death Dis..

[74] Chen J.J., Shen H.C., Rivera Rosado L.A., Zhang Y., Di X., Zhang B. (2012). Mislocalization of death receptors correlates with cellular resistance to their cognate ligands in human breast cancer cells. Oncotarget.

[75] Huang P., Liao R., Chen X., Wu X., Li X., Wang Y., Cao Q., Dong C. (2020). Nuclear translocation of PLSCR1 activates STAT1 signaling in basal-like breast cancer. Theranostics.

[76] Young T.M., Wang Q., Pe’ery T., Mathews M.B. (2003). The human I-mfa domain-containing protein, HIC, interacts with cyclin T1 and modulates P-TEFb-dependent transcription. Mol. Cell Biol..

[77] Thebault S., Mesnard J.M. (2001). How the sequestration of a protein interferes with its mechanism of action: Example of a new family of proteins characterized by a particular cysteine-rich carboxy-terminal domain involved in gene expression regulation. Curr. Protein Pept. Sci..

[78] Ikeda A., Shahid S., Blumberg B.R., Suzuki M., Bartlett J.D. (2019). ADAM10 is Expressed by Ameloblasts, Cleaves the RELT TNF Receptor Extracellular Domain and Facilitates Enamel Development. Sci. Rep..

[79] Bossen C., Ingold K., Tardivel A., Bodmer J.L., Gaide O., Hertig S., Ambrose C., Tschopp J., Schneider P. (2006). Interactions of tumor necrosis factor (TNF) and TNF receptor family members in the mouse and human. J. Biol. Chem..

[80] Vandevoorde V., Haegeman G., Fiers W. (1997). Induced expression of trimerized intracellular domains of the human tumor necrosis factor (TNF) p55 receptor elicits TNF effects. J. Cell Biol..

[81] He X.L., Garcia K.C. (2004). Structure of nerve growth factor complexed with the shared neurotrophin receptor p75. Science.

[82] Geerts D., Cusick J.K., Connelly L. (2020). Editorial: The Tumor Necrosis Factor Superfamily: An Increasing Role in Breast Cancer. Front. Oncol..

[83] Rivas M.A., Carnevale R.P., Proietti C.J., Rosemblit C., Beguelin W., Salatino M., Charreau E.H., Frahm I., Sapia S., Brouckaert P. (2008). TNF α acting on TNFR1 promotes breast cancer growth via p42/P44 MAPK, JNK, Akt and NF-kappa B-dependent pathways. Exp. Cell Res..

