# Supplementary material for: RELT Is Upregulated in Breast Cancer and Induces Death in Breast Cancer Cells
Source: Biomedicines. 2024 Nov 22;12(12):2667. doi: 10.3390/biomedicines12122667 (PMC11727564; doi:10.3390/biomedicines12122667)
Supplement: Supplementary file 1 [file biomedicines-12-02667-s001.zip › biomedicines-3269636-supplementary.pdf]

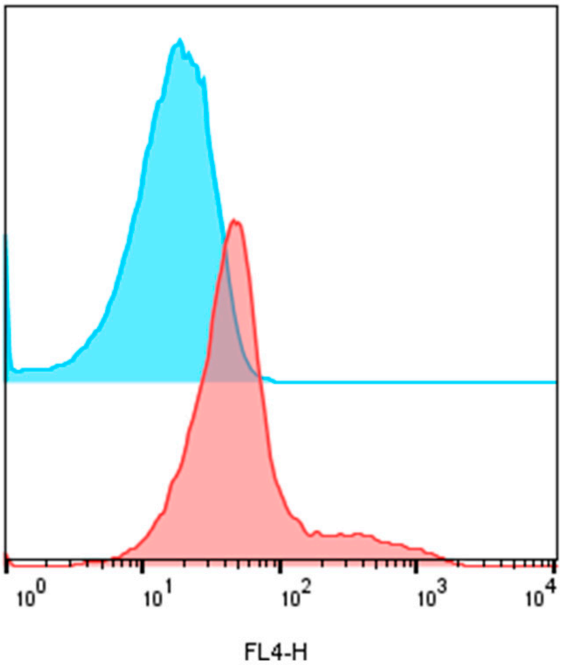

	Sample Name	Count	Mean : FL4-H
	Thp1 US.002	45673	22.8
	Thp1 S.001	45208	112

Thp1 cells

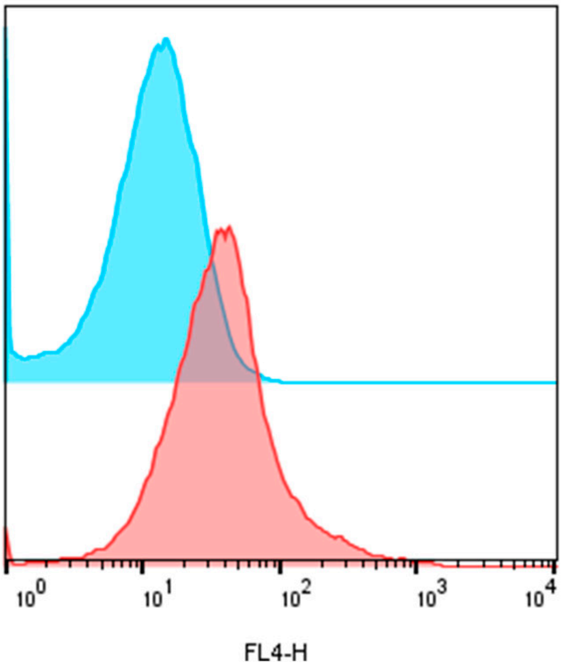

	Sample Name	Count	Mean : FL4-H
	RAJI US.014	42729	17.1
	RAJI S.013	42424	68.1

Raji cells

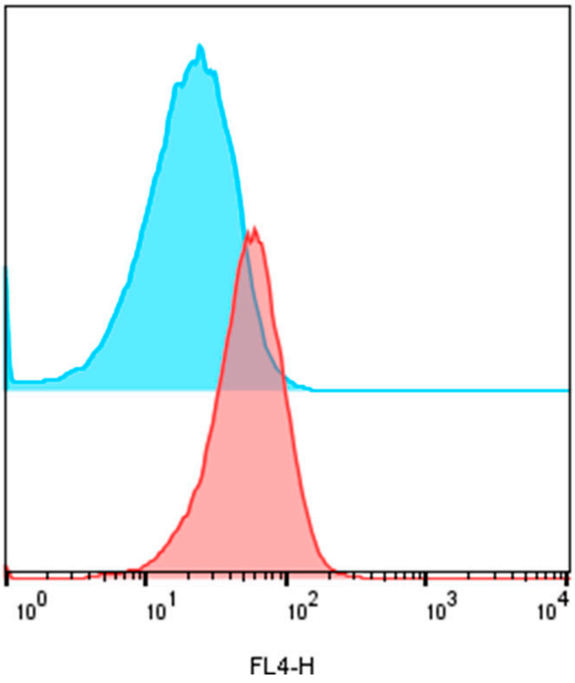

	Sample Name	Count	Mean : FL4-H
	Mcf7 US.004	47464	25.3
	Mcf7 S.003	47216	62.6

Mcf7 cells

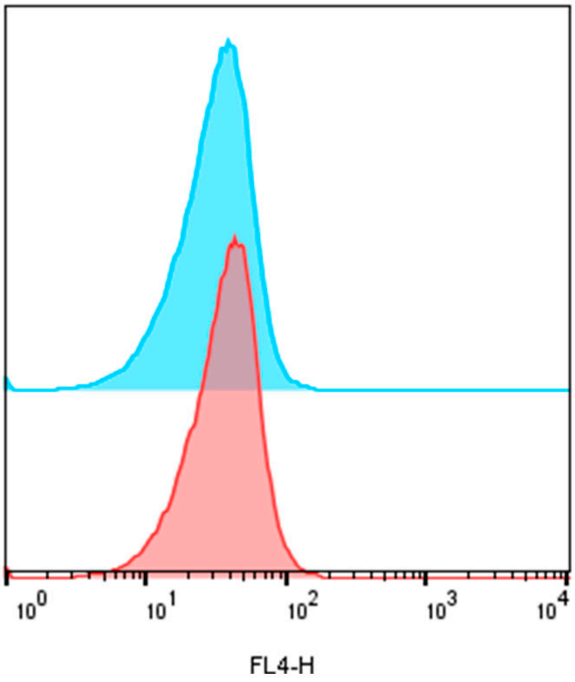

	Sample Name	Count	Mean : FL4-H
	358 US.006	47007	34.8
	358 S.005	47174	40.7

H358 cells

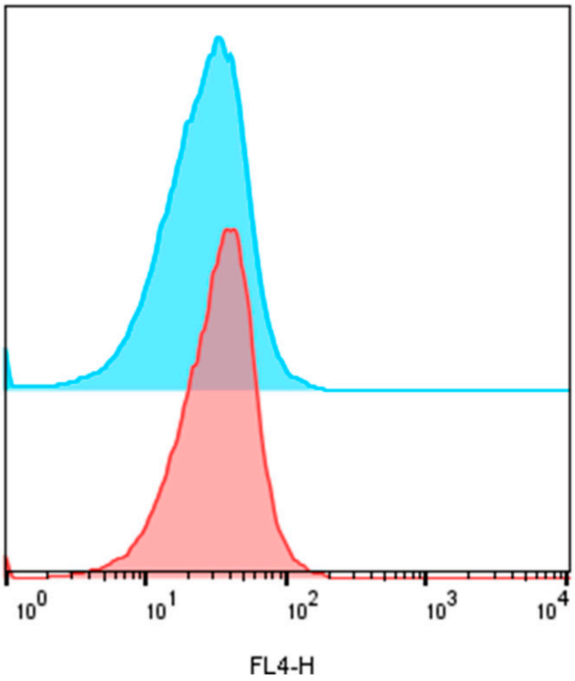

	Sample Name	Count	Mean : FL4-H
	231 US.010	46547	31.0
	231 S.009	46567	38.8

MDA-MB-231 cells

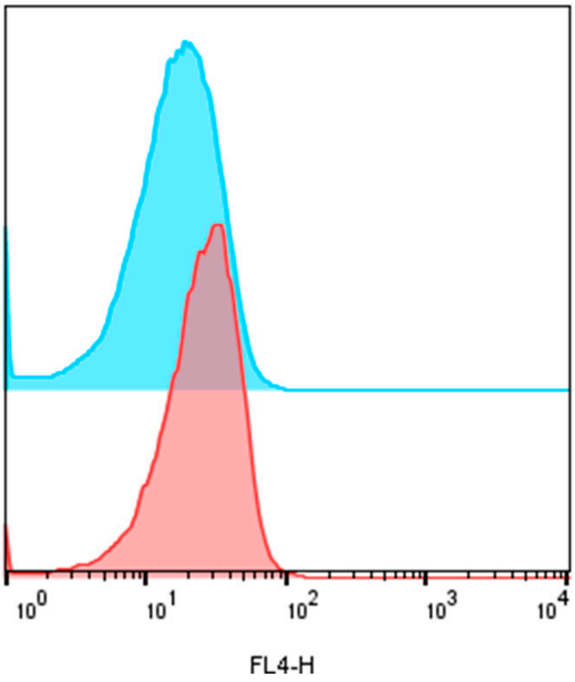

	Sample Name	Count	Mean : FL4-H
	293 US.008	46409	19.1
	293 S.007	46421	29.9

HEK-293 cells

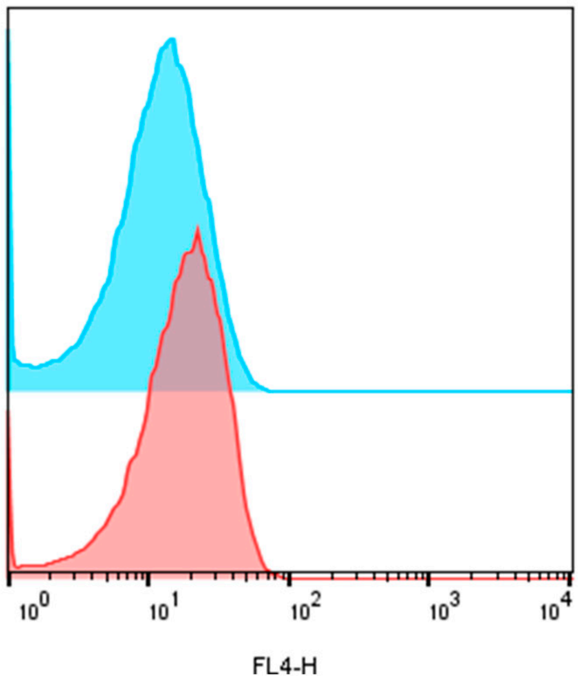

	Sample Name	Count	Mean : FL4-H
(blue)	Jurkat US.012	46992	15.2
(red)	Jurkat S.011	47167	20.7

Jurkat cells

Supplemental Figure S1. Comparison of unstained and stained flow cytometry histograms used to determine surface RELT expression in Fig. 1B. An anti-hRELT/TNFRSF19L Alexa Fluor 647 conjugated antibody was used to stain samples created from the indicated cell lines and analyzed by flow cytometry to quantify surface expression of RELT. Mean fluorescence index (MFI) was compared across all 7 cell lines using BD Biosciences FlowJo 10.

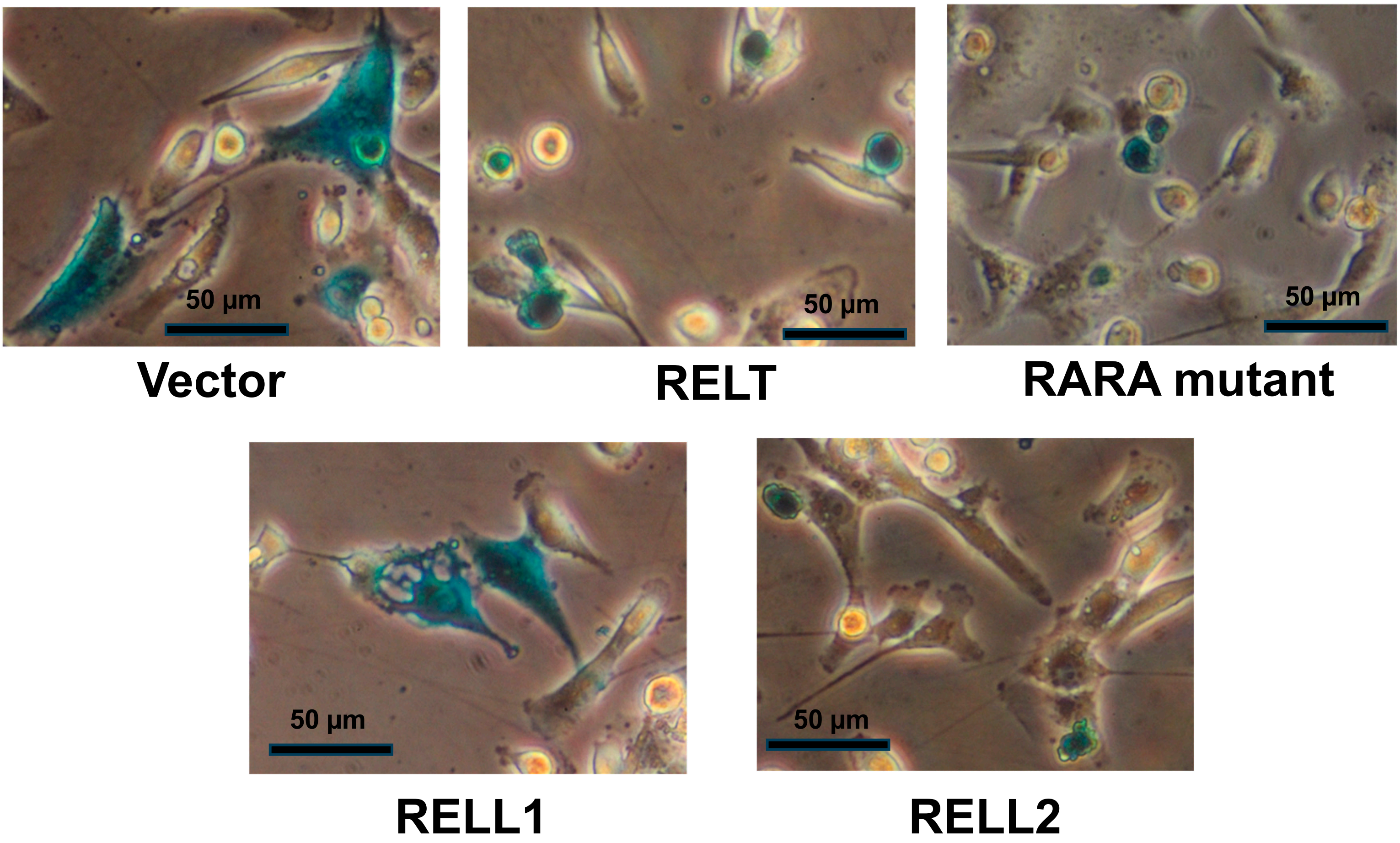

Supplemental Figure S2. X-gal morphology of 231 cells expressing RELT, RELL1, RELL2, and RARA mutant of RELT. 231 cells were transfected with the indicated constructs as well as an expression plasmid for β-galactosidase. Cells were stained with X-gal 48 hours after transfection as described in Materials and Methods. Representative images of cells transfected with either empty vector or an expression plasmid for RELL2 are shown at 60x magnification. Scale bar of 50 µm is indicated

Supplemental Figure S3.

RELT family members induce phosphatidylserine externalization in HEK-293 cells

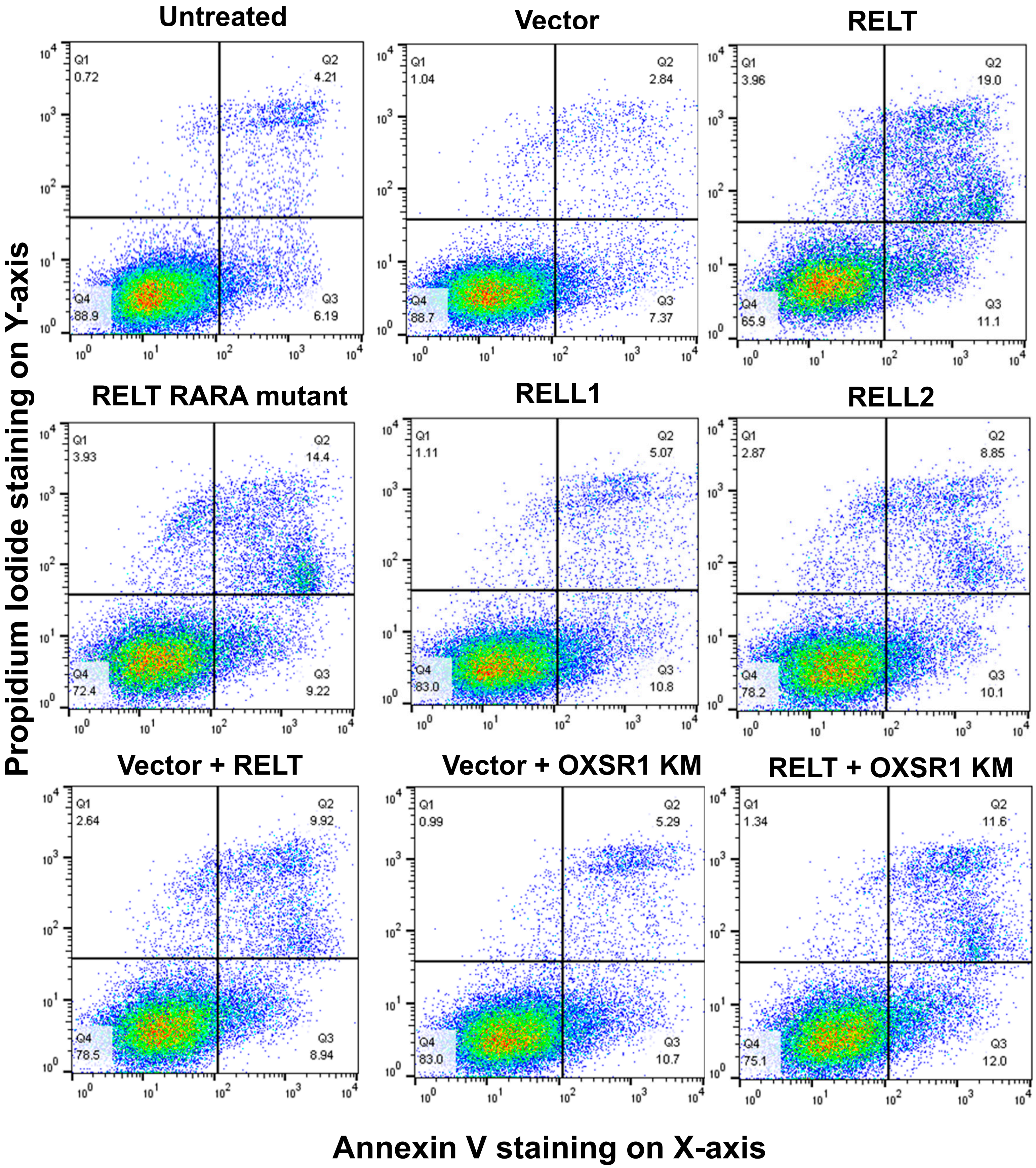

Supplemental Figure S3. RELT family members induce phosphatidylserine externalization in HEK-293 cells

293 cells were transfected with the indicated plasmids and flow cytometry was conducted 48 hours after transfection to determine phosphatidylserine externalization by Annexin V (AV) staining and cell death through Propidium Iodide (PI) staining as described in Materials and Methods. Cells were separated based on their staining for AV (X-axis) and PI (Y-axis) and the results were quantified based on single positive AV staining and double positive AV/PI staining. A representative of three separate experiments is shown.

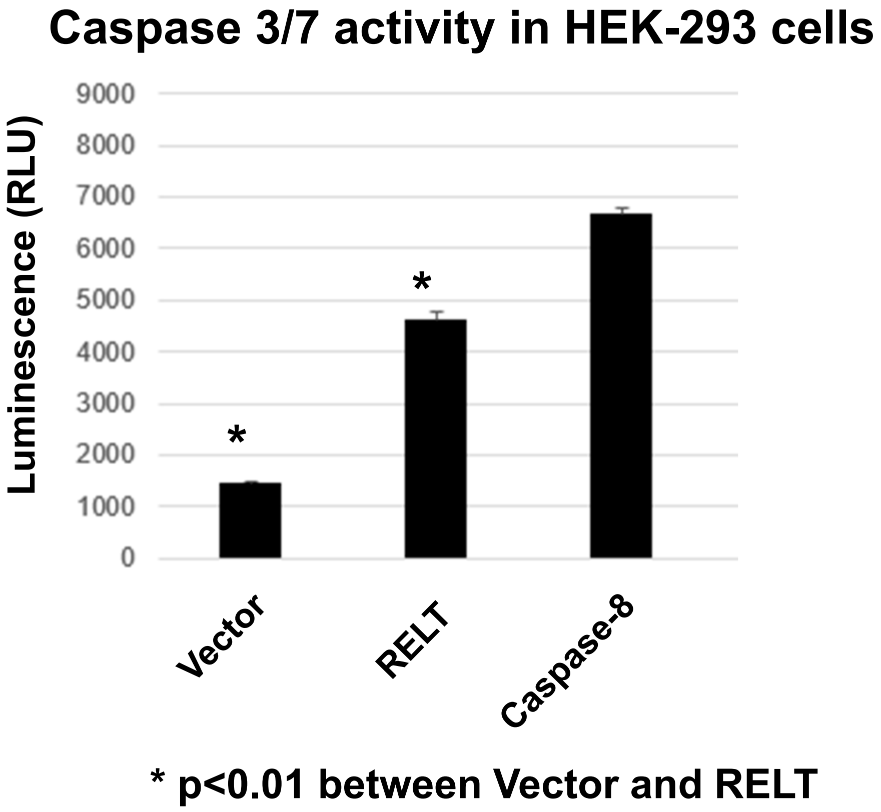

Supplemental Figure S4. RELT family members induce Caspase 3/7 activation in HEK-293 cells. 293 cells were transfected with the indicated constructs and a Caspase 3/7 luciferase assay was performed 48 hours after transfection as described in Materials and Methods. A representative of three experiments is shown. Significant values of P < 0.01 (*) are indicated.

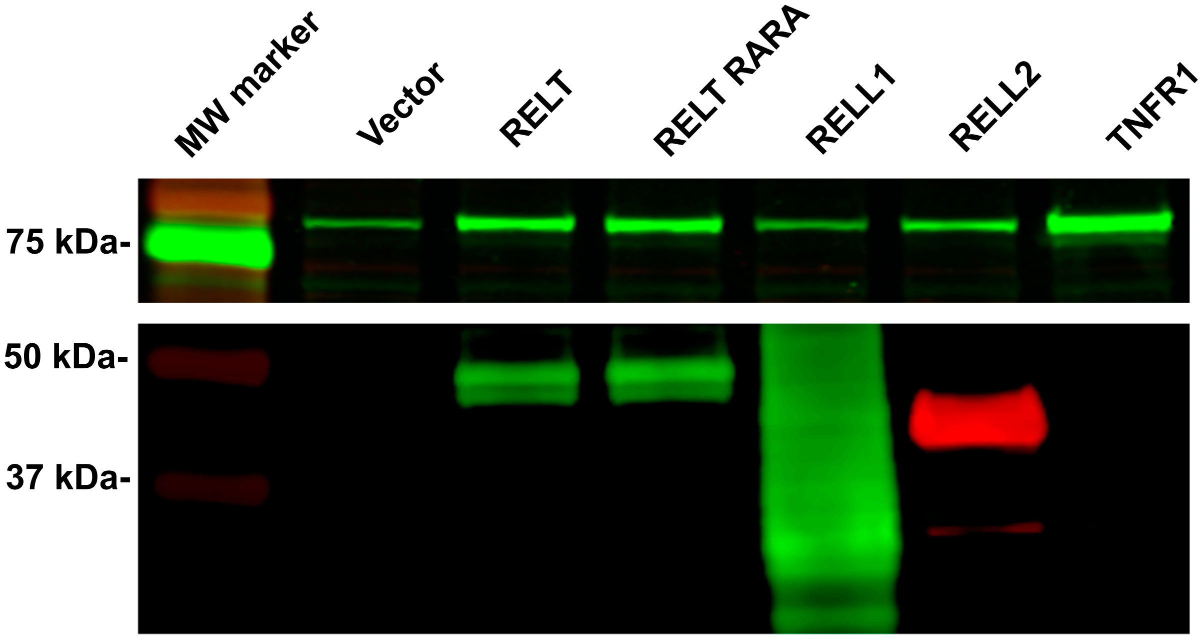

Supplemental Figure S5. RELT family members induce cleavage of PARP in HEK-293 cells. 293 cells were transfected with the indicated constructs and western blotting was performed 48 hours after transfection as described in Materials and Methods. A primary rabbit antibody (Ig) directed against cleaved PARP and an anti-rabbit IR 800 conjugated Ig were utilized to visualize cleaved PARP as green bands. The blot was reprobed with a primary rabbit anti-HA antibody followed by an anti-rabbit IR 800 conjugated antibody to visualize expected HA-epitope-tagged recombinant proteins (RELT, RELT RARA, and RELL1) as green bands. A mouse anti-Flag antibody followed by an anti-mouse IR 680 antibody was utilized to visualize Flag-tagged RELL2 as a red band. Molecular weight (MW) markers with sizes in kilodaltons (kDa) are indicated.

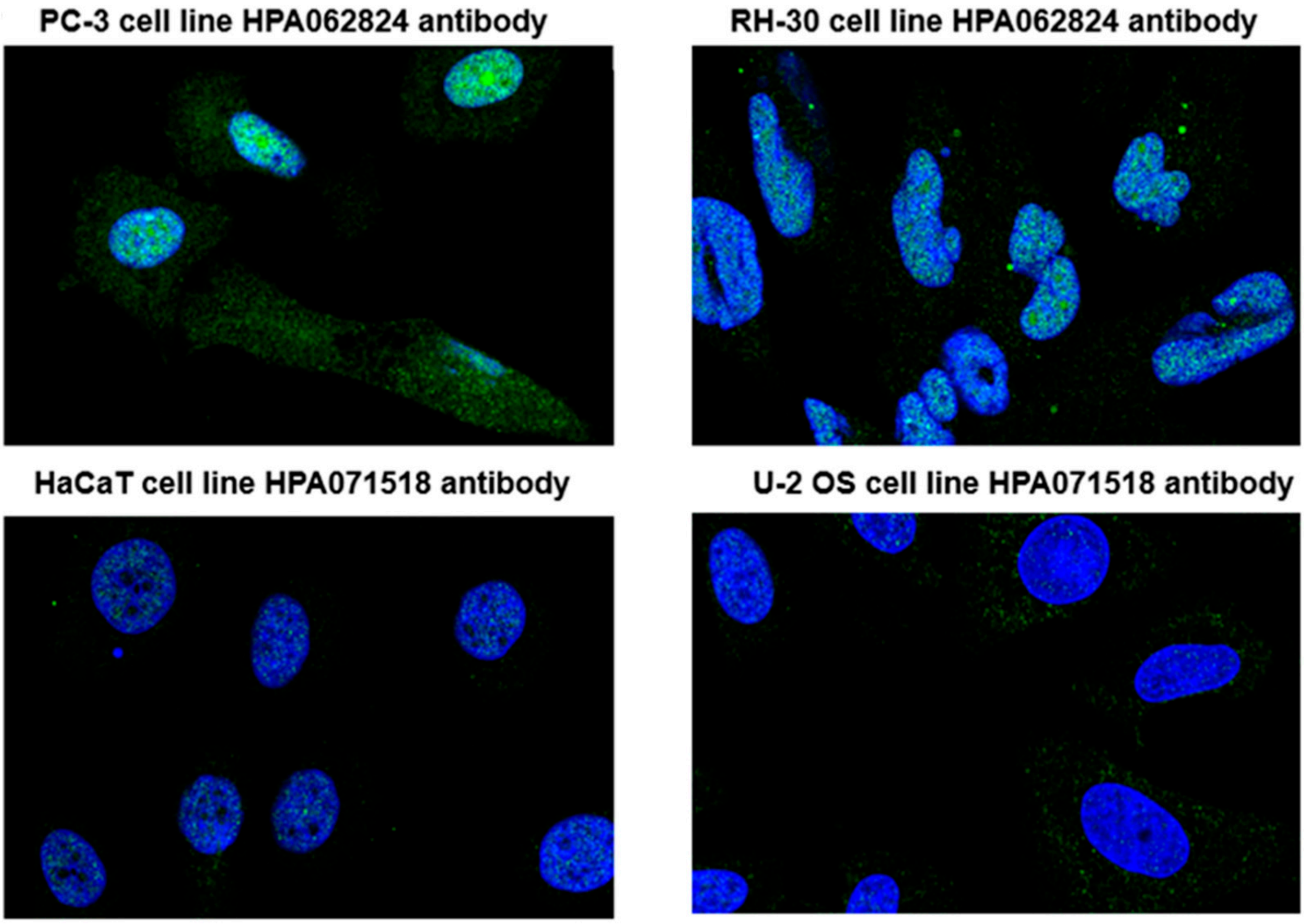

Supplemental Figure S6. Cellular localization of RELT as assessed by immunofluorescence (IF), Courtesy of Human Protein Atlas. https://v23.proteinatlas.org/ENSG00000054967-RELT/subcellular. Endogenous RELT localization reported by version 23 of the Human Protein Atlas in the following cell lines: PC-3 (prostatic adenocarcinoma), RH-30 (rhabdomyosarcoma), HaCaT (keratinocyte), and U-2 OS (osteosarcoma). IF was performed using either Sigma HPA062824, or with Sigma HPA071518, created with RELT residues 360-425 as immunogen.

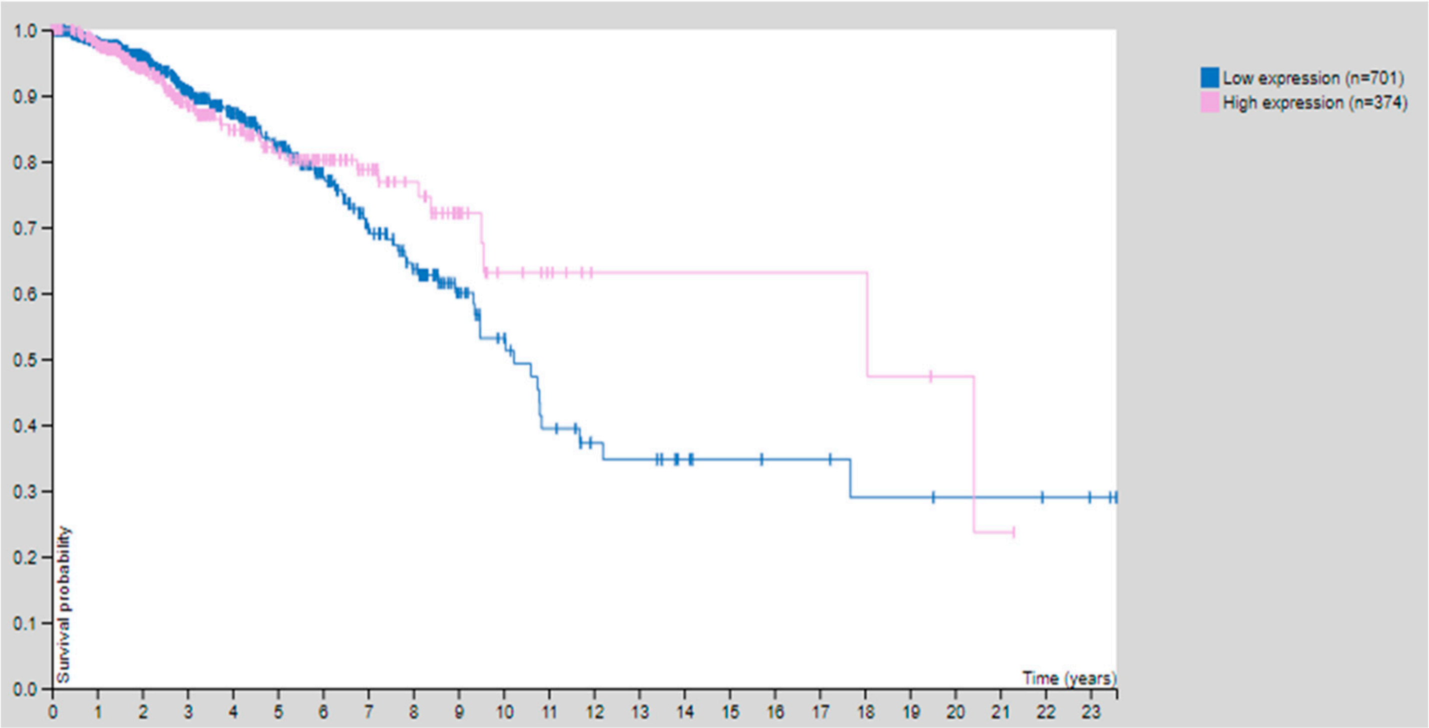

Supplemental Figure S7. Kaplan-Meier survival scatter plot for RELT and breast cancer, Courtesy of Human Protein Atlas, https://v23.proteinatlas.org/ENSG00000054967-RELT/pathology/breast+cancer. RELT is not a prognostic indicator for breast cancer.

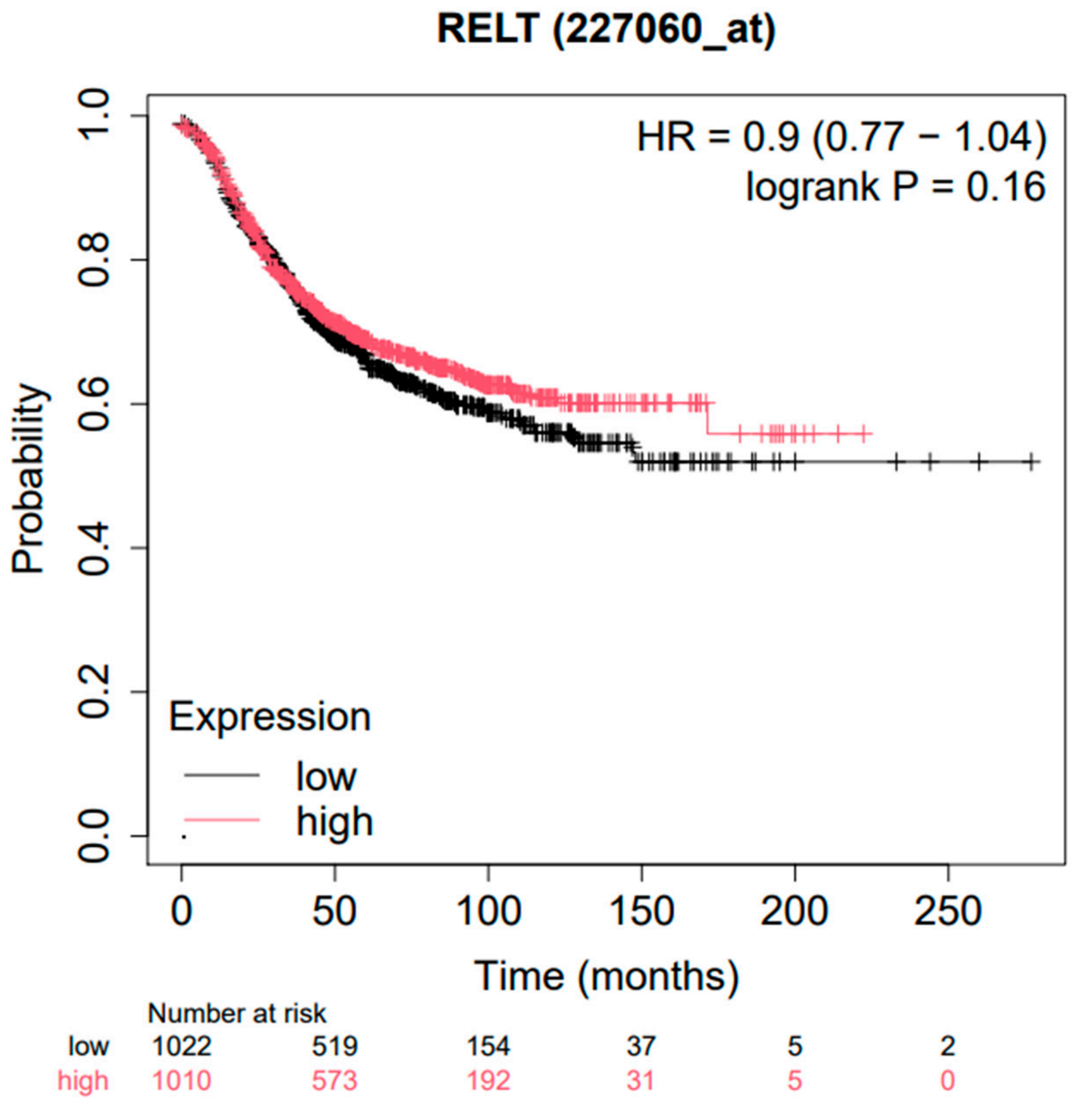

Supplemental Figure S8. Kaplan-Meier survival analysis of breast cancer patients stratified by RELT expression levels. The survival probability over time (months) is plotted for patients with high RELT expression (red line) and low RELT expression (black line).
